# Bis-Thiourea Chiral Sensor for the NMR Enantiodiscrimination of *N*-Acetyl and *N*-Trifluoroacetyl Amino Acid Derivatives

**DOI:** 10.1021/acs.joc.2c00814

**Published:** 2022-09-05

**Authors:** Alessandra Recchimurzo, Federica Balzano, Gloria Uccello Barretta, Luca Gherardi

**Affiliations:** Department of Chemistry and Industrial Chemistry, University of Pisa, via Moruzzi 13, 56124 Pisa, Italy

## Abstract

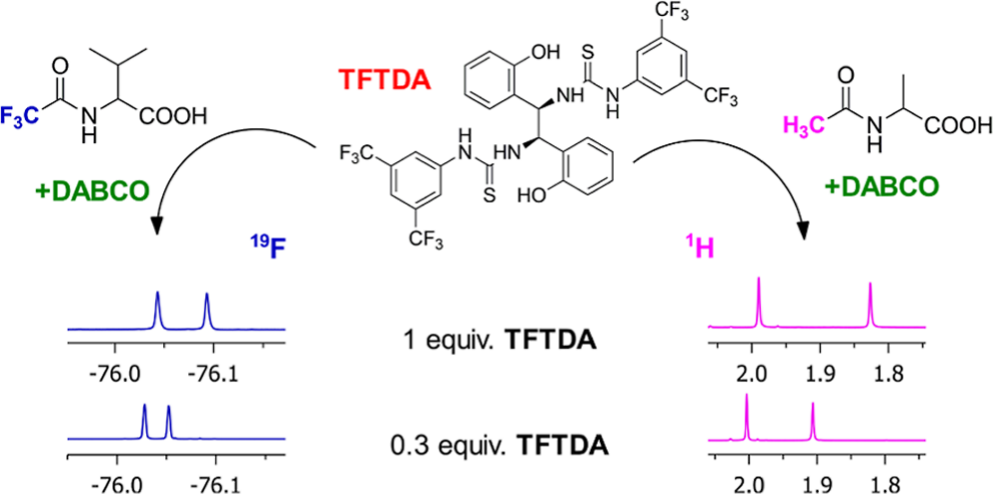

A C2-symmetrical bis-thiourea chiral solvating agent (CSA), **TFTDA**, for NMR spectroscopy has been obtained by reacting (1*R*,2*R*)-1,2-bis(2-hydroxyphenyl)ethylenediamine and 3,5-bis(trifluoromethyl)phenyl isothiocyanate. **TFTDA** shows remarkable propensity to enantiodiscriminate *N*-trifluoroacetyl (*N*-TFA) and *N*-acetyl (*N*-Ac) derivatives of amino acids with free carboxyl functions, with the co-presence of 1,4-diazabicyclo[2.2.2]octane (DABCO) as the third achiral additive, which is needed for substrate solubilization. **TFTDA** shows enhanced enantiodiscriminating efficiency in comparison with the corresponding monomeric counterpart, **TFTMA**, pointing out cooperativity between its two symmetrical entities. A wide range of amino acid derivatives have been efficiently enantiodiscriminated in CDCl_3_, with high enantioresolution quotients, which guarantee high quality in applications devoted to the quantification of enantiomers. High enantiodiscriminating efficiency is maintained also in diluted 5 mM conditions or in the presence of sub-stoichiometric amounts of CSA (0.3 equiv). The role of phenolic hydroxyls in the DABCO-mediated interaction mechanism between **TFTDA** and the two enantiomeric substrates has been pointed out by means of diffusion-ordered spectroscopy (DOSY) and rotating frame Overhauser effect spectroscopy (ROESY) experiments. A conformational model for both the CSA and its diastereomeric solvates formed with the two enantiomers of *N*-acetyl leucine has also been conceived on the basis of ROE data in order to give a chiral discrimination rationale.

## Introduction

Crucial role of chirality in life sciences has raised awareness of the scientific community to issues related to the availability of accurate, reproducible, and direct methods for the quantification of enantiomeric excesses (ees), among which chiral chromatography^1−4^ and NMR^5−11^ spectroscopy have attained huge popularity.

NMR methods of differentiation of enantiomeric mixtures are based on the use of suitable chiral auxiliaries able to induce anisochrony of enantiomer resonances by transferring them into a diastereomeric environment. Three main classes of chiral auxiliaries for NMR spectroscopy have been developed which are named chiral derivatizing agents (CDAs), chiral solvating agents (CSAs), and chiral lanthanide shift reagents (CLSRs). CSAs are particularly attractive from a practical point of view since any chemical derivatization is not required like in the case of CDAs, and they are simply mixed to the enantiomeric mixture under analysis directly into the NMR tube. CSAs are diamagnetic and hence do not produce unwanted line-broadening effects, like in the case of CLSRs, which can be detrimental for the accurate quantification of enantiomer resonances.

Several classes of CSAs have been proposed spanning from low molecular weight organic compounds to natural products or highly preorganized cyclic and acyclic structures.^6−10^ In the design of new CSAs for enantiomer differentiation by NMR, some fundamental requirements must be taken into consideration, that is, the presence of aromatic moieties able to exert, on the enantiomeric substrates, anisotropic effects and/or π–π interactions, leading to improved chemical shift differentiation, and the presence of hydrogen-bond donor/acceptor functions in order to stabilize the diastereomeric pairs formed in solution. Chiral thioureas, the potential of which as organocatalysts has been successfully demonstrated,^12−16^ have emerged also as CSAs^17−31^ for NMR endowed with remarkable enantiodiscriminating efficiency mainly due to their improved hydrogen-bond donating ability in comparison with the corresponding urea systems. Three main synthetic schemes have been followed in the design of bis-thiourea CSAs. The production of C2-symmetrical systems by reacting inexpensive chiral amines with thiophosgene,^26,28,30,31^ linking achiral diamines like 1,8-diaminoantracene to α-amino acid esters via thiourea groups,^24,26^ or the reaction of chiral diamines, mainly (1*S*,2*S*)-/(1*R*,2*R*)-1,2-diphenylethane-1,2-diamine^21,23,25,27^ or (1*S*,2*S*)-1,2-diaminocyclohexane,^17^ with 2 equiv of isothiocyanate has led to efficient CSAs. Effectiveness of the 3,5-bis(trifluoromethyl)phenyl moiety in boosting enantiodiscriminating efficiency both of amide and urea CSAs has also been widely proved.^23,25,27,32^

Interestingly, achiral additives like 4-dimethylaminopyridine (DMAP) or 1,4-diazabicyclo[2.2.2]octane (DABCO) play an active role in the chiral discrimination pathways, bridging together the CSA and enantiomers.^18−21,23,25,27^

**Figure 1:**
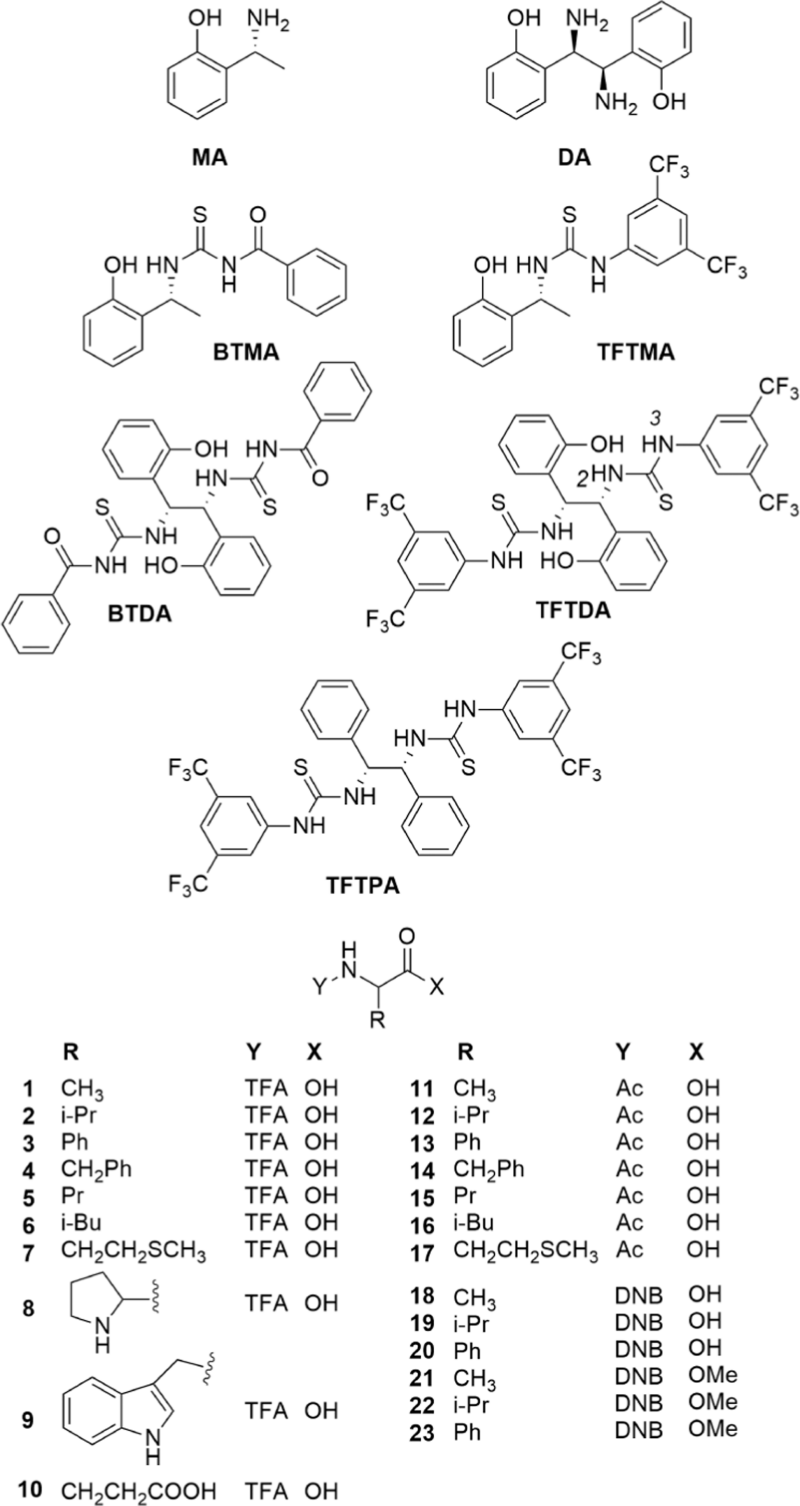
CSAs and amino acid derivative structures (TFA = trifluoroacetyl, Ac = acetyl, and DNB = 3,5-dinitrobenzoyl).

2-[(1*R*)-1-Aminoethyl]phenol (**MA**) and (1*R*,2*R*)-1,2-bis(2-hydroxyphenyl)ethylenediamine (**DA**) have been recently proposed^18,19^ as new chiral platforms for the production of mono- and bis-thiourea CSAs, with the idea of expanding the network of hydrogen bonds available for the interaction between the CSA and enantiomeric pairs, in virtue of the presence of acid phenolic hydroxyls. The reaction of **MA** and **DA** with benzoyl isothiocyanate^18,19^ led to the thiourea derivatives **BTMA** and **BTDA** (Figure 1), which were remarkably effective in the differentiation of NMR signals of *N*-3,5-dinitrobenzoyl (*N*-DNB) derivatives of amino acids both with free or derivatized carboxyl functions.^18,19^

Here, we evaluate the potential, as CSA, of a new C2-symmetric bis-thiourea system (**TFTDA**, Figure 1) based on **DA** (Figure 1), containing the 3,5-bis(trifluoromethyl)phenyl moiety, which is expected to affect acidity of thiourea NHs and hence complexing and enantiodiscriminating capabilities of the CSA. **TFTDA** has been employed in the differentiation of enantiomers of two different kinds of *N*-amino acid derivatives (Figure 1), that is, *N*-trifluoroacetyl (*N*-TFA, compounds **1–10**, Figure 1) and *N*-acetyl derivatives (*N*-Ac, compounds **11–17**, Figure 1), respectively, endowed with a fluorinated probe, suitable for the observation of enantiomers by ^19^F NMR, or an acetyl group, the signals of which are sharp singlets in a spectral region cleared of CSA signals. For comparison with previously reported **BTDA**,^18^ also *N*-DNB derivatives of amino acids (compounds **18–20**, Figure 1) and *N*-DNB derivatives of amino acid methyl esters (compounds **21–23**, Figure 1) were taken into consideration.

Relevance of the co-presence of 2-hydroxyphenyl and 3,5-bis(trifluoromethyl)phenyl moieties was pointed out by comparing the **TFTDA** enantiodiscriminating efficiency to that of previously reported chiral auxiliary **TFTPA** (Figure 1),^23,25,27^ having the same chemical structure, but devoid of phenolic hydroxyls.

Possible cooperativity of the two 3,5-bis(trifluoromethyl)phenylthiourea pendants of **TFTDA** was investigated by comparing **TFTDA** to its monomer analogous **TFTMA** (Figure 1). On considering that only few cases of CSAs able to produce efficient enantiodiscrimination in sub-stoichiometric conditions are reported,^22,32−37^ potential of **TFTDA** in this area was also evaluated.

To gain more information on the nature of the intermolecular interactions responsible for the chiral discrimination, NMR investigations have been carried out based on ROESY and DOSY experiments for the detection of through space dipolar interactions and translational diffusion, respectively.

## Results and Discussion

### ^1^H and ^19^F Enantiodiscrimination Experiments

**Scheme 1:**
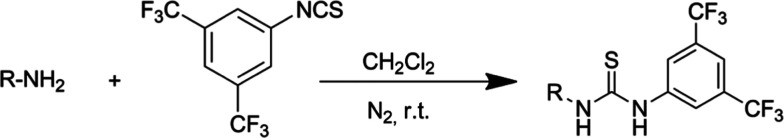
Synthesis of **TFTMA**, **TFTDA,** and **TFTPA**

Thiourea derivatives **TFTDA** and **TFTMA** were synthetized and obtained in a nearly quantitative yield, by reacting **DA** and **MA** with 2 or 1 equiv of 3,5-bis(trifluoromethyl)phenyl isothiocyanate (Scheme 1), respectively. **BTDA** and **TFTPA** were prepared in an analogous way. NMR characterization data are collected in the Experimental Section and Supporting Information.

Enantiodiscrimination experiments were performed by comparing the NMR spectra of binary equimolar mixtures **1–20**/DABCO and ternary mixtures **1–20**/DABCO/CSA in the suitable total concentration and molar ratio chiral substrate-to-CSA and solvent. The achiral additive DABCO, which has been demonstrated to be actively engaged in the chiral discrimination pathways,^18−21,23,25,27,32^ also acted as a solubility promoter for amino acid derivatives with poor solubility in the organic solvents considered in this investigation (CDCl_3_ and C_6_D_6_). The analysis of *N*-3,5-dinitrobenzoylamino acid methyl esters (**21–23**) did not require the presence of DABCO.^18,19^ The enantiodiscriminating efficiency was evaluated by measuring the chemical shift nonequivalence (ΔΔδ = |Δδ_R_ – Δδ_S_| = |δ_R_ – δ_S_|, ppm; where Δδ_R_ = δ_R_ – δ_F_, Δδ_S_ = δ_S_ – δ_F_, and δ_F_ is the chemical shift of the free substrate), that is, the difference of the chemical shifts of the corresponding nuclei of the two enantiomers δ_R_ and δ_S_ in the presence of the CSA.

**Figure 2:**
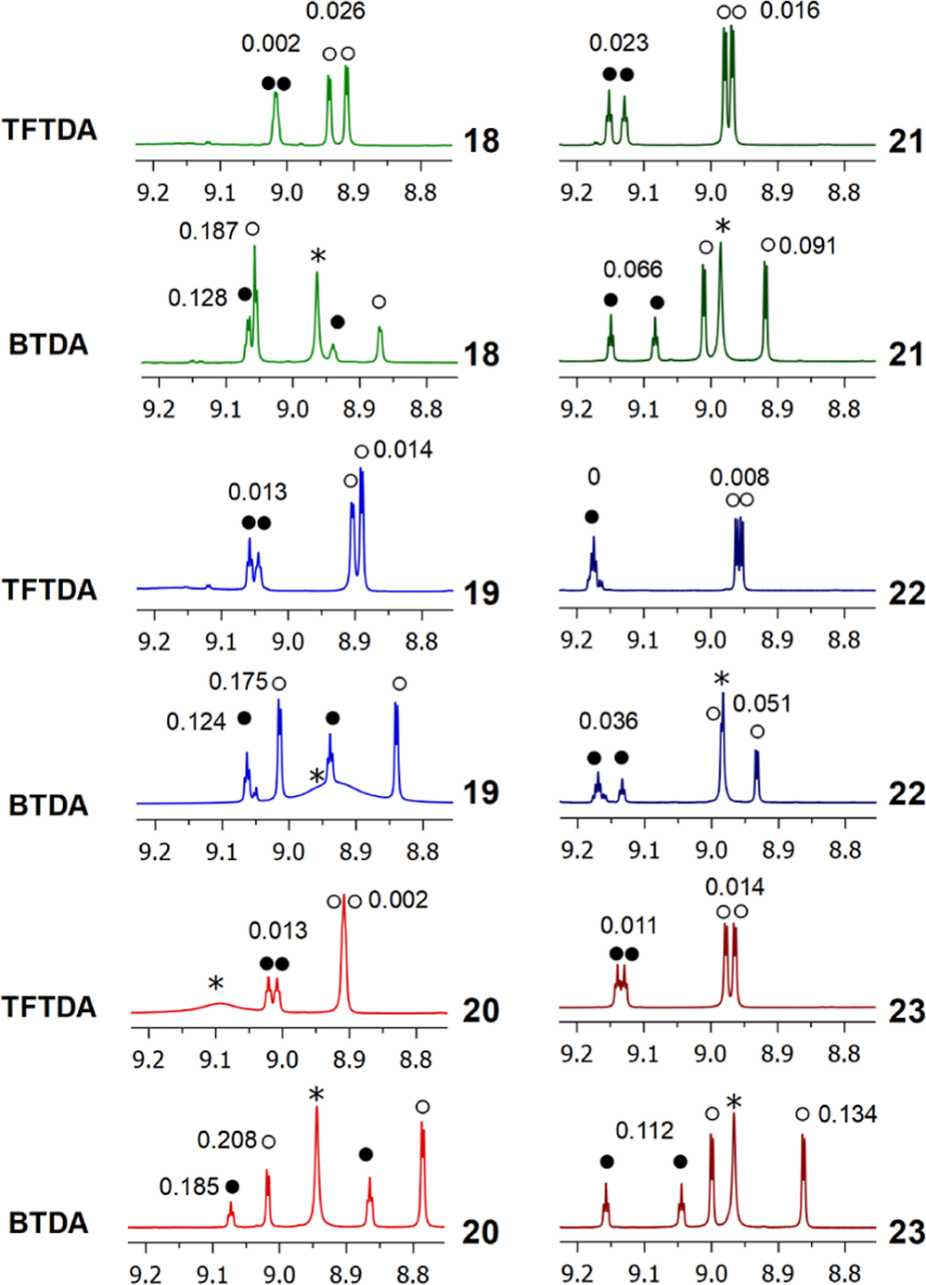
^1^H NMR (600 MHz, CDCl_3_, 25 °C) spectral regions corresponding to DNB resonances of **18–23** (15 mM)/DABCO/**BTDA** or **TFTDA** (1:1:1). ○ and ● indicate *ortho*- and *para*-DNB resonances, respectively. * indicates CSA resonances. Racemic or enantiomerically enriched samples of amino acid derivatives were analyzed. Nonequivalences in ppm are reported on resonances.

First, we looked for enantiodiscriminating peculiarities of bis-thiourea **TFTDA** as compared to previously reported **BTDA**. To this aim, we dealt with four different kinds of derivatives of alanine, valine, and phenylglycine, on considering derivatives with free carboxyl functions as *N*-TFA (**1–3**), *N*-Ac (**11–13**), *N*-DNB (**18–20**), and *N*-DNB-amino acid methyl esters (**21–23**). Superior capacity of **BTDA** to enantiodiscriminate amino acid derivatives endowed with *N*-DNB derivatizing groups was fully demonstrated in 15 mM equimolar mixtures, with even better performances for amino acid derivatives having free carboxyl functions (Figure 2, Table S1 in Supporting Information). As a general trend, *ortho*- and *para*-protons of the 3,5-dinitrophenyl moiety of the two enantiomers of phenylglycine, valine, and alanine underwent high differentiation up to 0.208 ppm, as in the case of *ortho*-protons of **20**. Nonequivalences measured in the mixtures containing derivatives **18–23** and **TFTDA** were instead remarkably lower (<0.026 ppm, Table S1 in Supporting Information).

**Figure 3:**
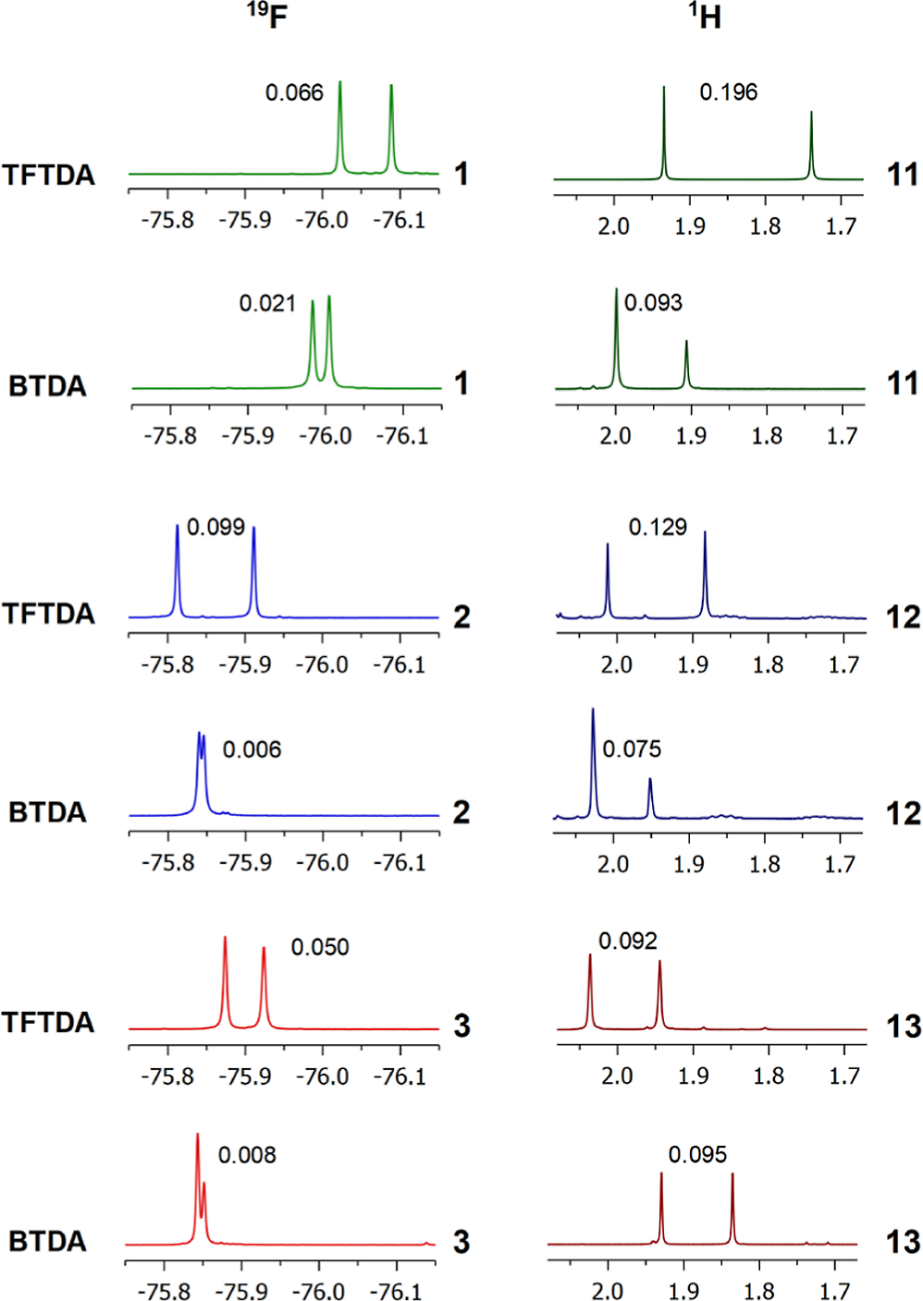
^19^F NMR (564 MHz, CDCl_3_, 25 °C) spectral regions corresponding to CF_3_ resonances of **1–3** (15 mM) and ^1^H NMR (600 MHz, CDCl_3_, 25 °C) spectral regions corresponding to acetyl resonances of **11–13** (15 mM) in the presence of 1 equiv of DABCO and **BTDA** or **TFTDA**. Racemic or enantiomerically enriched samples of amino acid derivatives were analyzed. Nonequivalences in ppm are reported on resonances.

The reverse is true regarding *N*-TFA derivatives **1–3** which were remarkably better enantiodiscriminated by **TFTDA** than they were by **BTDA** (Figure 3, Table S1 in Supporting Information). As a matter of fact, both NH and CH protons in the ^1^H NMR spectra and CF_3_ nuclei in the ^19^F spectra showed nonequivalences ranging from 0.012 to 0.096 ppm and from 0.050 to 0.099 ppm, respectively. The highest values measured in the presence of **BTDA** were 0.033 ppm for the NH proton of **3** and 0.021 ppm for the fluorine nuclei of **1**, that is, remarkably lower.

Similarly, in the case of NH and acetyl protons of **11–13**, higher nonequivalences were measured in the presence of **TFTDA** than those obtained in the presence of **BTDA** (Figure 3, Table S1 in Supporting Information). The sole exception was **13**, that is, the *N*-Ac derivative of phenylglycine, which gave very similar nonequivalences for the acetyl protons in the presence of **BTDA** or **TFTDA**. Anyway, **TFTDA** induced remarkably higher nonequivalences on NH (0.130 ppm) and CH (0.106 ppm) than those measured in the presence of **BTDA** (0.040 and 0.016 ppm for NH and CH, respectively).

To definitely highlight the peculiarities of **TFTDA** in the panorama of bis-thiourea systems, we compared **TFTDA** enantiodifferentiation of **6** and **16** with that of a very similar CSA (**TFTPA**),^27^ devoid of phenolic hydroxyls (Table 1). **TFTDA** allowed to attain better enantiomer differentiation: twofold increase of nonequivalence for CF_3_ of *N*-TFA leucine (**6**) and about 30-fold for acetyl of *N*-Ac leucine (**16**).

**Table 1:** ^1^H (600 MHz) and ^19^F (564 MHz) Nonequivalences (ΔΔδ = |δ_R_ – δ_S_|, ppm; CDCl_3_, 25 °C) for **6** and **16** (15 mM) in Equimolar Mixtures with **TFTDA** or **TFTPA** and in the Presence of 1 equiv of DABCO

	**TFTDA**	**TFTPA**
sub	CF_3_/Ac	CF_3_/Ac
**6**	0.090	0.047
**16**	0.190	0.006

The in-depth analysis of the enantiodiscrimination data requires a further comment which deals with the comparison between nonequivalence (ΔΔδ) and enantioresolution quotient (*E*) data,^38^ which also takes into account the average linewidth, making aware of the fact that high nonequivalences do not guarantee the quality of enantiodifferentiation for enantiomer quantification. Three cases can be targeted, with partial (0 < *E* < 1), moderate (*E* ca. 1), and high (*E* ≫ 1) enantioresolution.

Coming back to our data, we can observe that enantioresolution quotient *E* (Table S1 in Supporting Information) is largely greater than 1 for protons belonging to the derivatizing motif, thus guaranteeing a very high quality of enantiodifferentiation: E ranges from 2 to 13.9 for protons of *N*-DNB in the mixture with **BTDA** and becomes very high for *N*-Ac (13.3 < *E* < 28.0) and *N*-TFA (7 < *E* < 13.6) amino acid derivatives in the mixture with **TFTDA** due to differentiation of sharp singlets produced by acetyl and CF_3_ moieties. As a matter of fact, the very high nonequivalences of 0.208 and 0.185 ppm, respectively, measured for the *ortho*- (doublet) and *para*- (triplet) protons of **20** in its mixture with **BTDA** (Figure 2) corresponded to the remarkable *E*s of 13.9 and 8.4. On the other hand, for the acetyl protons of **12** in its mixture with **TFTDA** (Figure 3), an enantioresolution quotient of 18.4 was calculated, even though the nonequivalence was lower, that is, 0.129 ppm. The same is true for the fluorine resonances of *N*-TFA amino acid derivatives, sharp singlets of which allow, as an example, us to obtain an enantioresolution quotient of 13.6 for a nonequivalence magnitude of 0.099 ppm, as measured in the case of the mixture **2**/**TFTDA**/DABCO.

Ultimately, the possibility to detect nonequivalences on probe moieties of the amino acid derivatives, which produce sharp singlets, guarantees accurate enantiomer quantification even when enantiomer differentiations, that is, nonequivalences, are lower.

**Figure 4:**
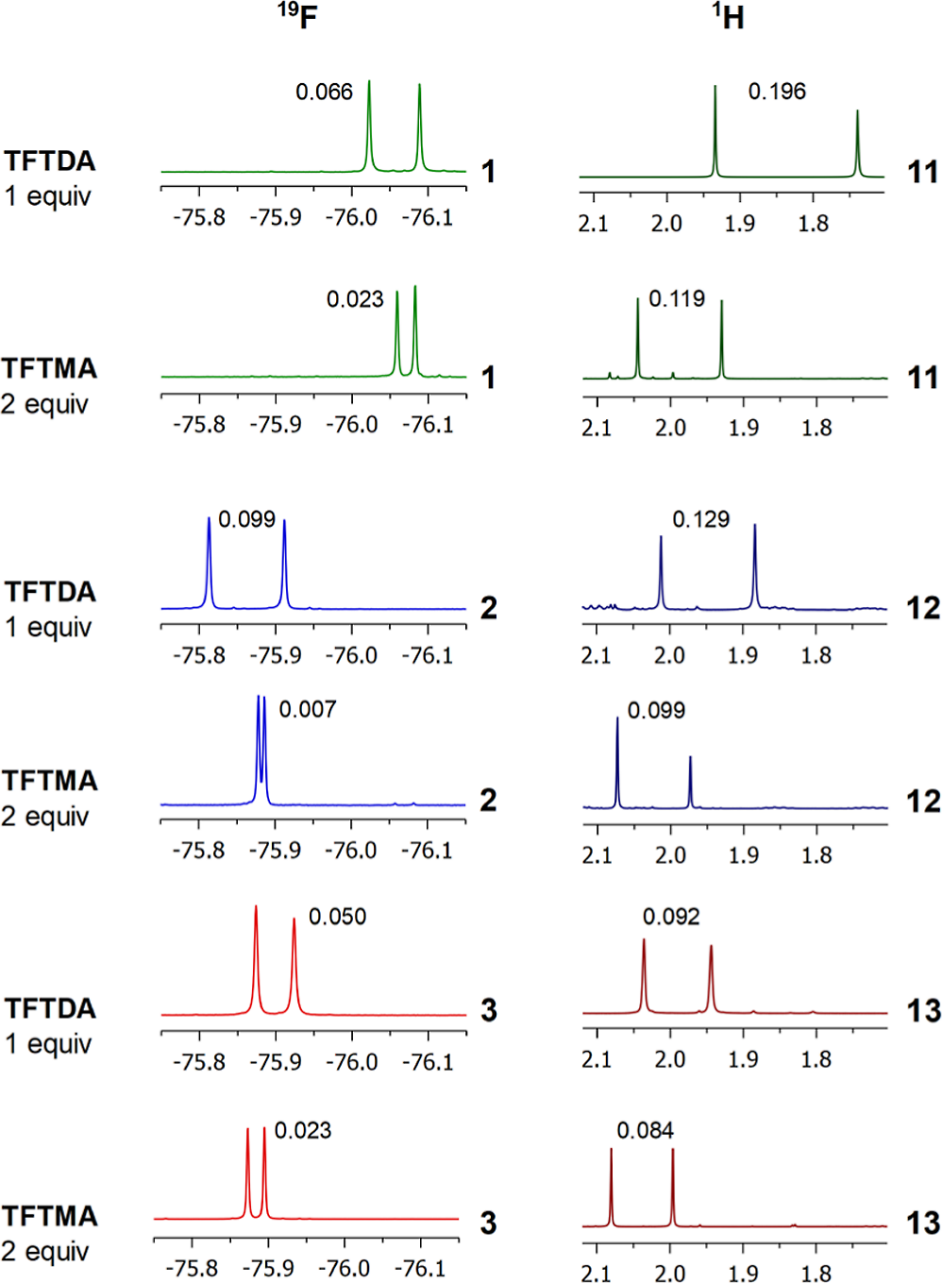
^19^F NMR (564 MHz, CDCl_3_, 25 °C) spectral regions corresponding to CF_3_ resonances of **1–3** (15 mM) and ^1^H NMR (600 MHz, CDCl_3_, 25 °C) spectral regions corresponding to acetyl resonances of **11–13** (15 mM) in the presence of 1 equiv of DABCO and 1 equiv of **TFTDA** or 2 equiv of **TFTMA**. Racemic or enantiomerically enriched samples of amino acid derivatives were analyzed. Nonequivalences in ppm are reported on resonances.

Once pointed out the remarkable enantiodiscriminating efficiency of **TFTDA** toward *N*-Ac and *N*-TFA amino acids, dimeric **TFTDA** and monomeric **TFTMA** (Figure 4, Tables S2 and S3 in Supporting Information) were compared in the enantiodiscrimination of the three representative *N*-TFA derivatives **1–3** and *N*-Ac derivatives **11–13**. Not only nonequivalences of 15 mM equimolar mixtures were remarkably lower in the presence of 1 equiv of the monomer CSA, **TFTMA**, in comparison with dimeric **TFTDA**, but also adding a further equivalent of **TFTMA** did not allow us to get the same nonequivalence magnitude found in the 15 mM equimolar mixture containing dimeric **TFTDA** (Figure 4).

For example, fluorine signals of the two enantiomers of **1** were differentiated to be 0.018 and 0.066 ppm in the mixtures containing equimolar amounts of **TFTMA** and **TFTDA**, respectively (Table S2 in Supporting Information). Nonequivalence induced by 2 equiv of **TFTMA** increased up to 0.023 ppm, which was lower than that measured in the equimolar mixture containing **TFTDA** (Figure 2). Therefore, cooperativity can be envisaged between the two thiourea arms of **TFTDA**, acting in concert, rather than independently, in the stabilization of the complexes formed with the enantiomeric pairs. Accordingly, on the basis of Job’s plot (Figure S1 in Supporting Information), the 1-to-1 stoichiometric ratio was found for the two complexes formed by **TFTDA** and the two enantiomers of the *N*-acetyl derivative of leucine **16**. An eventual independent interaction of the **TFTDA** arms would have favored a 1-to-2 CSA to the chiral substrate stoichiometric ratio.

**Figure 5:**
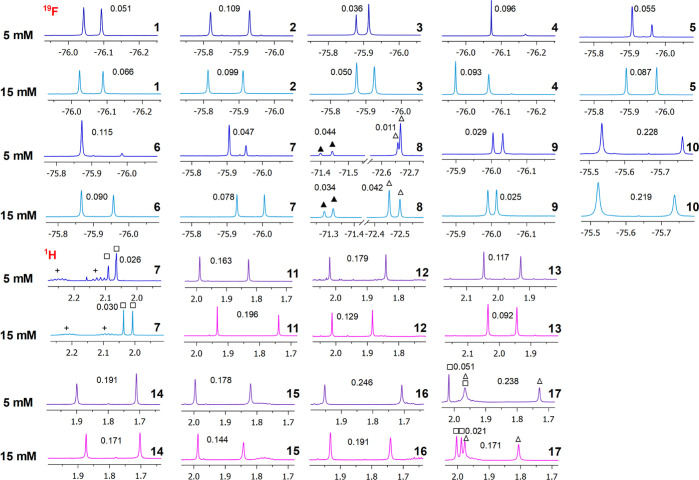
^19^F NMR (564 MHz, CDCl_3_, 25 °C) spectral regions corresponding to CF_3_ (△) of **1–10** (15 and 5 mM) and ^1^H NMR (600 MHz, CDCl_3_, 25 °C) spectral regions corresponding to acetyl (△) of **11–17** (15 and 5 mM) in the presence of 1 equiv of DABCO (2 equiv for **10**) and **TFTDA**. ▲ indicates the *syn* stereoisomer of **8**. □ indicates methylthio resonances of **7** and **17,** and + indicates side chain protons of **7**. Racemic or enantiomerically enriched samples of amino acid derivatives were analyzed. Nonequivalences in ppm are reported on resonances.

To deal with enantiodiscriminating versatility of **TFTDA**, besides compounds **1–3** and **11–13**, already discussed, we extended our analysis to *N*-TFA derivatives **4–10** and *N*-Ac ones **14–17** (Figure 1). Equimolar 15 mM mixtures were analyzed in CDCl_3_ in the presence of 1 equiv of DABCO (Table 2 and Figure 5 showing CF_3_ and acetyl resonances).

**Table 2:** ^1^H (600 MHz) and ^19^F (564 MHz) Nonequivalences (ΔΔδ = |δ_R_ – δ_S_|, ppm; CDCl_3_, 25 °C) and Enantioresolution Quotients (*E*, in Parentheses) for **1–17** (15 mM or 5 mM) in Equimolar Mixtures with **TFTDA** and in the Presence of 1 equiv (for **1–9** and **11–17**) or 2 equiv (for **10**) of DABCO

	15 mM		5 mM	
sub	NH	CF_3_/Ac	NH	CF_3_/Ac
**1**	0.054 (0.9)	0.066 (8.1)	0.038 (0.7)	0.051 (7.1)
**2**	0.048 (0.8)	0.099 (13.6)	0.040 (0.7)	0.109 (15.1)
**3**	0.082 (1.5)	0.050 (7.0)	0.014 (0.3)	0.036 (4.9)
**4**	0.222 (3.9)	0.093 (13.5)	0.216 (4.1)	0.096 (13.5)
**5**		0.087 (12.6)	0.064 (1.2)	0.055 (7.6)
**6**	0.110 (1.9)	0.090 (9.7)	0.105 (1.9)	0.115 (16.1)
**7**^a^		0.078 (11.2)		0.047 (6.8)
**8**		0.042 (6.1), 0.034 (4.9)		0.011 (1.5), 0.044 (6.1)
**9**		0.025 (3.6)		0.029 (4.0)
**10**	1.249 (21.9)	0.219 (31.3)	1.391 (25.7)	0.228 (31.4)
**11**	0.152 (2.3)	0.196 (28.0)	0.108 (1.7)	0.163 (25.7)
**12**	0.066 (1.0)	0.129 (18.4)	0.088 (1.4)	0.179 (28.4)
**13**	0.130 (2.0)	0.092 (13.3)	0.129 (2.1)	0.117 (18.8)
**14**	0.166 (2.5)	0.171 (24.4)	0.158 (2.5)	0.191 (30.3)
**15**	0.112 (1.7)	0.144 (20.6)	0.118 (1.8)	0.178 (28.3)
**16**	0.156 (2.4)	0.191 (27.3)	0.157 (2.5)	0.246 (39.0)
**17**^b^	0.057 (0.9)	0.171 (24.4)	0.024 (0.5)	0.238 (38.2)

a Nonequivalences of 0.030 ppm (*E* = 4.8) and 0.026 ppm (*E* = 3.5) were measured for the MeS group at 15 and 5 mM, respectively.

b Nonequivalences of 0.021 ppm (*E* = 2.3) and 0.051 ppm (*E* = 6.3) were measured for the MeS group at 15 and 5 mM, respectively.

^19^F nonequivalences ranged from 0.025 ppm for tryptophan derivative **9** to the very high value of 0.219 ppm measured in the case of glutamic acid derivative **10**, solubilization of which, however, required the presence of 2 equiv of DABCO. Interestingly, very high nonequivalences were also detected for the ^1^H resonances of NH protons, which reached the value of 1.249 ppm for glutamic acid derivative **10** (Table 2). Nonequivalences detected for the acetyl protons of derivatives **11–17** were once again very high, in the range 0.092 ppm for **13** up to 0.196 ppm for **11** (Table 2, Figure 5). In almost all cases, a very high enantiodiscriminating efficiency is guaranteed with no concern about the quality of enantiodiscrimination, always with enantioresolution quotients largely greater than 1 (Table 2). It is noteworthy that in the case of proline derivative **8**, nonequivalence was detected for the possible *syn*- and *anti*-stereoisomers. The methylthio group of methionine derivatives **7** and **17** were even efficiently enantiodifferentiated (Figure 5, Table 2).

The use of less polar C_6_D_6_ would be expected to bring about an enhancement of enantiomer differentiation since this solvent, compared to CDCl_3_, should interfere to a less extent with the stabilization of diastereomeric pairs. Surprisingly, nonequivalences measured for the CF_3_ and acetyl nuclei of amino acid derivatives were lower in C_6_D_6_ than those detected in CDCl_3_ (Table S4, Figures S2–S4 in Supporting Information). Exceptions were **11** and **16** with very similar values in the two solvents and **4** and **8** with higher values in C_6_D_6_. CH and NH moieties were better differentiated in the most apolar solvent, even though this was not a general trend, as no differentiation at all was found for the NH protons of the two enantiomers of **2–4**. Even in cases of very high differentiation of NH protons, however, direct detection of their resonances can be made difficult by unwanted superimpositions with CSA resonances (Figure S5 in Supporting Information), and to extract their signals, 1D TOCSY experiments must be performed by selective perturbation at the frequencies of their *J*-connected CH protons.

Going on with the use of CDCl_3_ as the solvent enabling us to detect enhanced differentiation of the probe signals, acetyl and trifluoroacetyl, we evaluated ability of **TFTDA** to maintain detectable nonequivalences also in more diluted solution. To this aim, progressively diluted equimolar solutions of **TFTDA** and *N*-Ac derivatives **11–17** were analyzed (Figure S6, Table S5 in Supporting Information). Unexpected results were obtained since dilution did not seem to affect significantly the enantiomer differentiation. Nonequivalence of acetyl protons of **11** started from a high value of 0.196 ppm at 15 mM and underwent a small decrease to a value of 0.163 ppm at 5 mM. For derivatives **12–17,** dilution even brought a nonequivalence increase up to 39% (Figure S6 in Supporting Information).

Analogously, nonequivalences measured in the fluorine spectra of *N*-TFA derivatives seemed to be scarcely responsive to dilution with fluctuating results, that is, almost unchanged nonequivalences for **2**, **4**, **9**, and **10**, a decrease for **1**, **3**, **5**, and **7**, and an increase for **6** and **8** (Figures 5 and S7 in Supporting Information).

Explaining such a kind of behavior is not trivial because dilution should cause a decrease of enantiomer bound fractions and, consequently, a decrease of nonequivalence. Observing even an opposite trend suggests the co-presence of simultaneous complexation equilibria. Therefore, we took into consideration the occurrence of eventual self-aggregation processes involving the CSA, by comparing its NMR spectra at 15 and 5 mM (Figure S8 and Table S6 in Supporting Information). In spite of the very limited concentration range, the chemical shift of the NH(3) proton of **TFTDA,** adjacent to the 3,5-bis(trifluoromethyl)phenyl moiety, was low-frequency shifted for 0.46 ppm in the 5 mM solution, compared to the 15 mM solution, witnessing its involvement in intermolecular hydrogen bonds reasonably due to self-aggregation, which is less favored in more diluted solution. The same trend was found for NH(2) but with a minor response to the concentration change (0.03 ppm): this last proton lies, in fact, in the inner part of the CSA structure. To confirm such a kind of hypothesis, we performed DOSY^39^ experiments in progressively diluted solution (15–3 mM) to measure the diffusion coefficient of the CSA (Table 3).

**Table 3:** ^1^H NMR (600 MHz, CDCl_3_, 25 °C) Diffusion Coefficient (*D* × 10^10^ m^2^ s^–1^) of **TFTDA** at Different Concentrations

	15 mM	5 mM	3 mM
*D* × 10^10^ (m^2^/s)	5.16 ± 0.06	5.72 ± 0.03	6.19 ± 0.06

In the spherical approximation, diffusion coefficient *D* depends on the hydrodynamic radius (*r*_H_), based on the Stokes–Einstein equation (eq 1)
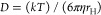
1where *k* is the Boltzmann constant, *T* is the absolute temperature, and η is the dynamic viscosity of the solution.

In the presence of the self-aggregation processes, the diffusion coefficient is the weighted average of its value in the monomer (*D*_M_) and self-aggregated species (*D*_SA_) (eq 2)
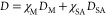
2where χ_M_ and χ_SA_ are the monomer and self-aggregated species molar fractions, and *D*_M_ and *D*_SA_ are the diffusion coefficients of the monomer and self-aggregated species, respectively.

A decrease of the diffusion coefficient, proportional to the molar fraction of the self-aggregated species, is expected in more concentrated solutions due to the increased molecular sizes of the self-aggregated species.

The CSA diffusion coefficient in 3 mM solution was equal to 6.19 × 10^–10^ m^2^ s^–1^ and decreased to a value of 5.16 × 10^–10^ m^2^ s^–1^ in 15 mM solution, confirming a contribution to the measured diffusion coefficient coming from a self-aggregated form of the CSA. On this basis, we can conclude that in more diluted solution, a greater amount of the non-aggregated form of the CSA is present in solution, available for the interaction with the two enantiomers of the chiral substrates and hence for their enantiodifferentiation.

In light of the above-said peculiar behavior, we also investigated the possibility to operate in CSA sub-stoichiometric conditions, which, in principle, should better discourage self-aggregation phenomena in favor of heteroaggregation of the CSA with enantiomers of amino acid derivatives. Therefore, equimolar 5 mM solutions of the *N*-TFA derivatives **1–3** and *N*-Ac derivatives **11–13** were compared to mixtures at the same substrate concentration (5 mM) but in the presence of 0.3 equiv of the CSA (Table 4 and Figure S9 in Supporting Information). In sub-stoichiometric conditions, nonequivalences both of CF_3_ nuclei of *N*-TFA and of COCH_3_ protons of *N*-Ac derivatives underwent a maximum reduction of nonequivalence by one-quarter but still giving very high enantioresolution quotients, ranging from 3.5 to 4.9 for **1–3** and from 4.8 to 15.4 for **11–13**. Therefore, sub-stoichiometric conditions, which are quite unusual for CSAs,^22,32−37^ can be suitably exploited, also employing the new dimeric thiourea CSA, **TFTDA**.

**Table 4:** ^1^H (600 MHz) and ^19^F (564 MHz) Nonequivalences (ΔΔδ = |δ_R_ – δ_S_|, ppm; CDCl_3_, 25 °C) and Enantioresolution Quotients (*E*, in Parentheses) of CF_3_ for **1–3** and of Ac for **11–13** (5 mM) in the Presence of 1 equiv of DABCO and 0.3 or 1 equiv of **TFTDA**

	ΔΔδ (ppm)	
	0.3 equiv **TFTDA**	1 equiv **TFTDA**
**1**	0.025 (3.5)	0.051 (7.1)
**2**	0.035 (4.9)	0.109 (15.1)
**3**	0.032 (4.4)	0.036 (4.9)
**11**	0.097 (15.4)	0.163 (25.7)
**12**	0.082 (13.1)	0.179 (28.4)
**13**	0.030 (4.8)	0.117 (18.8)

**Figure 6:**
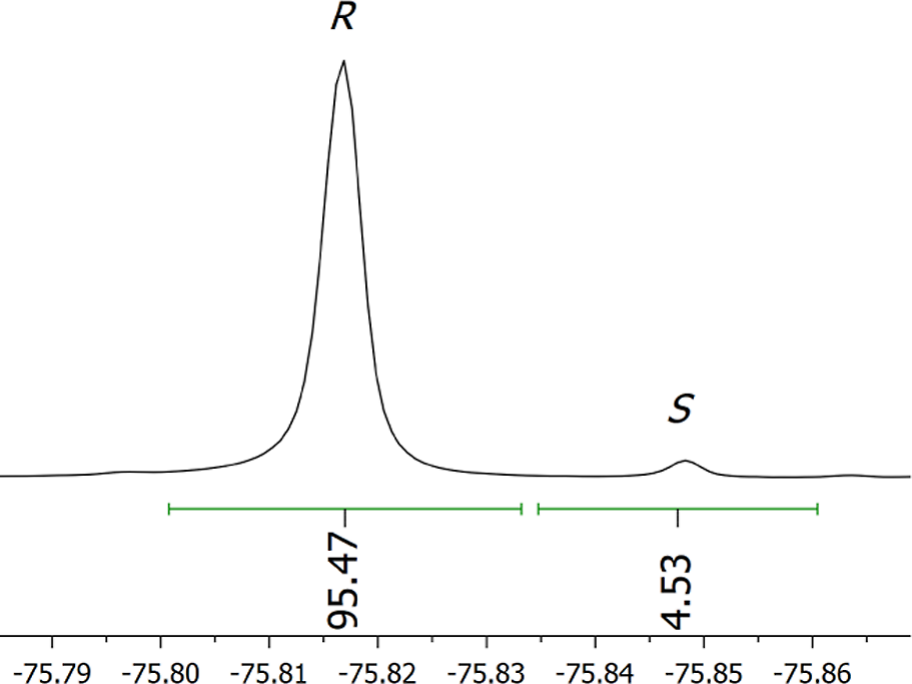
^19^F NMR (564 MHz, CDCl_3_, 25 °C) spectral region corresponding to CF_3_ resonances of enantiomerically enriched (ee +90, *R*/*S* = 95:5) **3** (15 mM) in the presence of 1 equiv of DABCO and 1 equiv of **TFTDA**.

Suitability of **TFTDA** as the CSA for real ee determination was evaluated in enantiomerically enriched samples of **3** (+90% e.e.) in equimolar 15 mM solution (Figure 6). The relationship between gravimetric and NMR data was excellent, with the absolute error within ±1%.

### NMR Investigation of Chiral Recognition Processes

**Figure 7:**
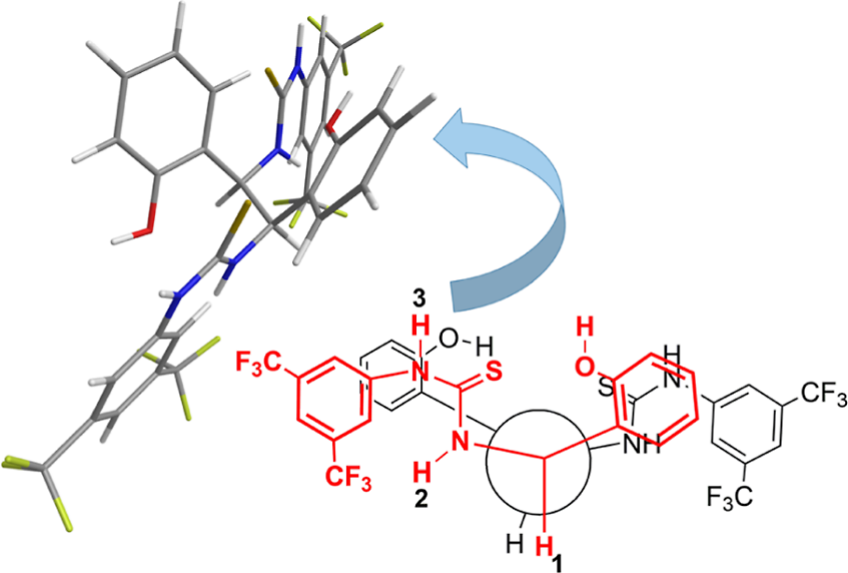
Schematic 3D representation of **TFTDA** according to NMR data and its Newman projection.

First, we looked for a conformational model for the CSA, **TFTDA**, even though this task was difficult to achieve in consideration of the symmetry of the system. Despite this, selected ROEs (Figure S10 in Supporting Information) allowed us to impose some spatial proximity constraints, which led to the schematic representation in Figure 7. In detail, the intense dipole–dipole interaction detected between the methine protons CH1 and the adjacent NH(2) protons of the thiourea moiety (Figure S10 in Supporting Information) allowed us to locate these two protons in a cisoid arrangement. NH(2) protons showed the expected reciprocal ROE effect at the frequency of CH1 protons but no effect at all on NH(3) (Figure S10 in Supporting Information). Therefore, the two NHs are in reciprocal transoid positions. Accordingly, a ROE between NH(2) and *ortho* protons of the 3,5-bis(trifluomethyl)phenyl moiety bound to NH(3) was detected (Figure S10 in Supporting Information). Therefore, ROE data support a *syn*–*anti* conformation for **TFTDA** in CDCl_3_ solution. Interestingly relevant ROE effects were detected between the phenolic protons and protons of the 3,5-bis(trifluoromethyl)phenyl groups (Figure S10 in Supporting Information) to witness their spatial proximity, which reasonably is due to attractive π–π interactions between the 2-hydroxyphenyl and 3,5-bis(trifluoromethyl)phenyl groups.

Reasonably, electron-withdrawing CF_3_ groups not only produce the expected enhancement of acidity of thiourea moieties, but also enhance the π-acidic character of the aromatic moieties they are bound to, thus favoring intramolecular attractive π–π interactions with the phenolic rings (as supported by ROE measurements) at the expense of intermolecular interactions. On this basis, the lower enantiodiscriminating efficiency of **TFTDA** with respect to **BTDA** toward substrates bearing *N*-3,5-DNB derivatizing groups can be rationalized.

As a next step, we went deeply into the role of DABCO in the enantiodifferentiation phenomena caused by **TFTDA**, beyond its solubilizing effect on the amino acid derivatives having free carboxyl functions. To this aim, first, we compared the diffusion coefficients of DABCO (15 mM) in CDCl_3_ as a pure compound and in equimolar binary mixtures DABCO/**TFTDA**. To better understand the role of DABCO in the presence of phenolic protons, we also analyzed DABCO diffusion behavior in the presence of **TFTPA** (1 equiv), devoid of phenolic OHs.

The diffusion coefficient of pure DABCO was 14.5 × 10^–10^ m^2^ s^–1^ and remarkably lowered to the value of 7.1 × 10^–10^ m^2^ s^–1^ in the presence of **TFTDA**, by contrast in the presence of **TFTPA,** its value slightly decreased to 13.9 × 10^–10^ m^2^ s^–1^ (Table S7 in Supporting Information).

The above results can be rationalized on the hypothesis that phenolic hydroxyls are themselves involved in the DABCO-mediated tight network of hydrogen bond interactions. To support the above conclusion, 1D ROESY experiments were carried out by selective perturbation at the frequency of DABCO protons in the mixture containing equimolar amounts of DABCO (15 mM) and **TFTDA** (Figure S11a in Supporting Information). Very intense dipolar interactions were detected at the frequency of phenolic protons, together with lower intensity ROEs at the frequency of H4 and H5 protons of the 3,5-bis(trifluoromethyl)phenyl moiety in the DABCO/**TFTDA** binary mixture (Figure S11 in Supporting Information).

To go deeper into the interaction mechanism responsible for the chiral discrimination, we focused on the equimolar ternary mixtures containing DABCO (15 mM), **TFTDA,** and the two enantiomers of *N*-acetyl leucine (**16**), where *N*-acetyl groups underwent remarkable enantiomeric differentiation in the presence of the CSA. 1D ROESY experiments were performed to detect intermolecular dipolar interactions and hence define proximity constraints between the two enantiomeric substrates and the CSA. The methoxy group of (*S*)-**16** produced comparable intermolecular ROEs on the protons H9 and H4 belonging to the 2-hydroxyphenyl and 3,5-bis(trifluoromethyl)phenyl moieties, respectively (Figure S12 in Supporting Information). Alkyl protons of its isobutyl group showed selectivity for the protons of the fluorinated aromatic ring (Figure S13 in Supporting Information). The reverse was found for (*R*)-**16**, with its acetyl moiety in closer proximity of the 3,5-bis(trifluoromethyl)phenyl group of the CSA (Figure S12 in Supporting Information) and almost equivalent ROEs at the frequency of 2-hydroxyphenyl and 3,5-bis(trifluoromethyl)phenyl groups (Figure S13 in Supporting Information) for alkyl protons of the isobutyl group. Interestingly, the methine protons of the two enantiomers of **16** gave very similar ROEs on the two aromatic rings of the CSA (Figure S14 in Supporting Information). In the ternary mixtures, DABCO protons produce dipolar interactions with amino acid protons and π-acid aromatic moiety of the CSA and its *o*-hydroxyphenyl moiety, which means that the base lies in between all of these groups (Figure S11b,c in Supporting Information). **TFTDA** retains its free state *syn*–*anti* conformation in the presence of the enantiomers of *N*-Ac and *N*-TFA amino acid derivatives, as demonstrated by the detection of NH(2)-H4 ROE in the mixture **TFTDA**/substrate/DABCO (examples are reported in Figures S15 and S16 in Supporting Information). According to this bound state conformational preference, highly differentiated complexation shifts were detected for the two NH protons, that is, very low (0.05–0.09 ppm) for NH(2) and remarkably high (0.59–0.82 ppm) for NH(3) interacting with the enantiomeric substrates (Table S8 in Supporting Information). Even though unexpected, the interaction of thiourea receptors in their *syn*–*anti* conformation is already reported in the literature.^18,40−45^

**Figure 8:**
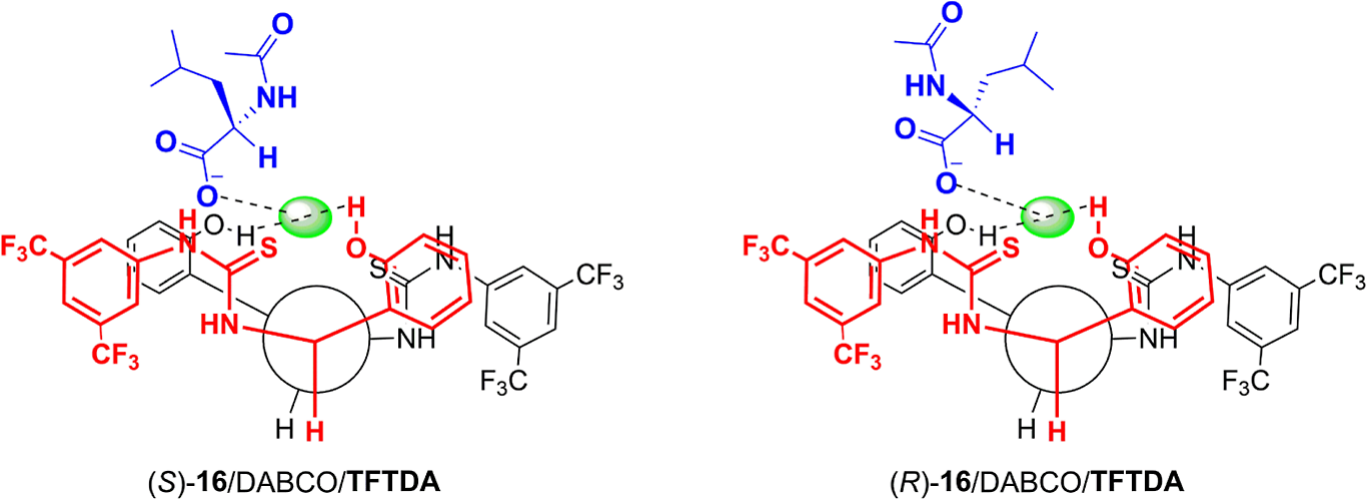
Schematic model of two diastereomeric complexes (*S*)-**16**/**TFTDA**/DABCO and (*R*)-**16**/**TFTDA**/DABCO.

Therefore, it can be concluded that the main stabilizing interactions are DABCO-mediated hydrogen bond interactions involving the thiourea moiety, NH(3) in particular, phenolic hydroxyls of the CSA, and the carboxyl function of the amino acid derivative. In this way, the two enantiomers always face the CSA from the same side and only an interchange between the two groups (acetyl *vs* alkyl) bound to the chiral center of the amino acid occurs, which is responsible for the chemical shift differentiation due to the anisotropic effects exerted by the aromatic moieties of the CSA. A schematic representation of the two diastereomeric solvates, which brings together all the information coming from ROEs and complexation shifts, is given in Figure 8.

Finally, we calculated the association constants for the two diastereomeric complexes. On the basis of the analysis of progressively diluted solution in the range from 15 to 2 mM, comparable values of 29.4 ± 3 and 29.6 ± 3 M^–1^ were calculated for (*R*)-**16**/**TFTDA** and (*S*)-**16**/**TFTDA**, respectively (Figure S17 in Supporting Information).

Therefore, it can be assessed that enantiomer differentiation in the NMR spectra is mainly due to anisotropic effects of aromatic moieties of the CSA, rather than thermodynamic differentiation of the two diastereomeric complexes.

## Conclusions

*N*-Derivatization of amino acids as *N*-trifluoroacetyl and *N*-acetyl derivatives makes possible the NMR differentiation of their enantiomers by detection of their fluorine signals and acetyl protons, respectively, both producing sharp signals ideal for accurate integration. Bis-thiourea **TFTDA** containing both 2-hydroxyphenyl and 3,5-bis(trifluoromethyl)phenyl moieties represents a CSA with high selectivity and enantiodiscriminating efficiency toward these two classes of amino acid derivatives. The synthesis both of the CSA and amino acid derivatives is very practical and does not require purification steps.

Very high nonequivalences are measured in 15 mM equimolar solution CSA/substrate/DABCO, which remain quite unaffected by dilution, at least up to 5 mM, thus allowing us to reduce the consumption of the CSA. Contrary to what was observed for the majority of CSAs reported in the literature, **TFTDA** has the unusual characteristic of producing efficient enantiodiscrimination also in the CSA sub-stoichiometric conditions. Ability of **TFTDA** to produce enantioresolution both in diluted and sub-stoichiometric conditions can be ascribed to its tendency to self-aggregate in solution, which is better inhibited both in diluted solutions or in the presence of enantiomeric substrate excesses.

**TFTDA** reasonably engages both its two lateral arms in the stabilization of the diastereomeric solvates formed with the two enantiomers of each amino acid derivative, as supported by comparison with the corresponding monomeric CSA and by the one-to-one complexation stoichiometric ratio. DABCO mediates the hydrogen bond interactions involving the carboxylate of the amino acid derivative, the thiourea, and phenolic moieties of the CSA.

Finally, **TFTDA** adopts a *syn*–*anti* conformation, even in the bound form, acting via a single NH hydrogen bond with the amino acid derivatives that can cooperatively form the hydrogen bond with the hydroxyls of the phenol moiety of the CSA.

## Experimental Section

### Materials

(1*R*,2*R*)-1,2-Bis(2-hydroxyphenyl)ethylenediamine (**DA**), 2-[(1*R*)-1-aminoethyl]phenol (**MA**), 3,5-bis(trifluoromethyl)phenyl isothiocyanate, and deuterated chloroform (CDCl_3_) were purchased from Aldrich and used without further purification. All *N*-Ac derivatives **11–17** were purchased from Alfa Aesar. Derivatives **1–10** and **18–23** were prepared, as described in refs (33) and (19), respectively. The NMR characterization is reported in ref (33). **BTDA** and **BTMA** were prepared, as described in ref (19).

### General Methods

^1^H, ^19^F, and ^13^C{^1^H} NMR measurements were carried out on a spectrometer operating at 600, 564, and 150 MHz for ^1^H, ^19^F, and ^13^C nuclei, respectively. The samples were analyzed in the C_6_D_6_ or CDCl_3_ solution, ^1^H and ^13^C chemical shifts are referred to tetramethylsilane (TMS) as the secondary reference standard, ^19^F chemical shifts are referred to trifluorotoluene as the external standard, and the temperature was controlled (±25 °C). For all of the 2D NMR spectra which were employed for the characterization of CSAs, the spectral width used was the minimum required in both dimensions. The gCOSY (gradient correlation spectroscopy) and TOCSY (total correlation spectroscopy) maps were recorded by using a relaxation delay of 1 s and 200 increments of 4 transients, each with 2K points. For TOCSY maps, a mixing time of 80 ms was set. The 1D-TOCSY spectra were recorded using a selective pulse, transients ranging from 128 to 256, a relaxation delay of 3 s, and a mixing time of 80 ms. The 2D-ROESY (rotating-frame Overhauser enhancement spectroscopy) maps were recorded by using a relaxation time of 3 s and a mixing time of 0.4 s; 256 increments of 16 transients of 2K-points each were collected. The 1D-ROESY spectra were recorded using a selective inversion pulse with transients ranging from 256 to 1024, a relaxation delay of 5 s, and a mixing time of 0.4 s. The gHSQC (gradient heteronuclear single quantum coherence) and gHMBC (gradient heteronuclear multiple bond correlation) spectra were recorded with a relaxation time of 1.2 s, 128–200 increments with 16 transients, each of 2K-points. The gHMBC experiments were optimized for a long-range coupling constant of 8 Hz. The assignment of ^1^H NMR and ^13^C{^1^H} NMR chemical shifts, reported below, is shown in Figures S18–S21 in Supporting Information. DOSY (diffusion-ordered spectroscopy) experiments were carried out using a stimulated echo sequence with self-compensating gradient schemes and 64 K data points. Typically, g was varied in 20 steps (2–32 transients each), and Δ and δ were optimized in order to obtain an approximately 90–95% decrease in the resonance intensity at the largest gradient amplitude. The baselines of all arrayed spectra were corrected prior to processing the data. After data acquisition, each FID was apodized with 1.0 Hz line broadening and Fourier transformed. The data were processed with DOSY macro (involving the determination of the resonance heights of all the signals above a pre-established threshold and the fitting of the decay curve for each resonance to a Gaussian function) to obtain pseudo two-dimensional spectra with NMR chemical shifts along one axis and calculated diffusion coefficients along the other.

### Synthesis **TFTMA**

3,5-Bis(trifluoromethyl)phenyl isothiocyanate (0.470 g, 1.70 mmol, 1 equiv) was added to **MA** (0.240 g, 1.70 mmol, 1 equiv) in CH_2_Cl_2_ (20 mL) at room temperature, under a nitrogen atmosphere. The reaction mixture was stirred at room temperature for 24 h and monitored by ^1^H NMR. The solvent was removed by evaporation under vacuum to afford chemically pure **TFTMA** in a nearly quantitative yield.

**TFTMA**. Straw yellow amorphous solid (0.684 g, 1.67 mmol, 98.5% yield) having mp 129–131 °C. [α]^23^_D_ = 38.4 (*c* = 0.5, CHCl_3_). ^1^H NMR (CDCl_3_, 600 MHz): δ 7.90 (s, 1H), 7.70 (s, 3H), 7.24 (d, 1H, *J* = 7.6 Hz), 7.20 (t, 1H, *J* = 7.6 Hz), 7.00 (d, 1H, *J* = 7.6 Hz), 6.95 (t, 1H, *J* = 7.6 Hz), 6.83 (d, 1H, *J* = 7.6 Hz), 6.12 (br s, 1H), 5.73 (s, 1H), 1.60 (d, 3H, *J* = 7.1 Hz). ^13^C{^1^H} NMR (CDCl_3_, 150 MHz): δ 178.9, 153.0, 138.5, 132.9 (q, *C*_CF_ = 33.7 Hz), 129.4, 128.0, 127.1, 123.8, 122.7 (q, *C*_CF_ = 273.8 Hz), 121.5, 119.4, 116.7, 51.9, 20.3. ^19^F NMR (CDCl_3_ 564 MHz): δ −63.04. Anal. Calcd for C_17_H_14_N_2_SOF_6_: C, 50.00; H, 3.46; N, 6.86. Found: C, 49.94; H, 3.51; N, 6.89.

### Synthesis **TFTDA**

3,5-Bis(trifluoromethyl)phenyl isothiocyanate (2.10 g, 7.58 mmol, 2 equiv) was added to **DA** (0.975 g, 3.79 mmol, 1 equiv) in CH_2_Cl_2_ (20 mL) at room temperature, under a nitrogen atmosphere. The reaction mixture was stirred at room temperature for 24 h and monitored by ^1^H NMR. The solvent was removed by evaporation under vacuum to afford chemically pure **TFTDA** in a nearly quantitative yield.

**TFTDA**. White amorphous solid (2.94 g, 3.74 mmol, 98.8% yield) having mp 169–172 °C. [α]^23^_D_ = −49.6 (*c* = 0.5, CHCl_3_). ^1^H NMR (CDCl_3_, 600 MHz): δ 8.37 (s, 2H), 7.93 (br s, 2H), 7.76 (s, 4H), 7.72 (s, 2H), 7.05 (t, 2H, *J* = 7.7 Hz), 6.95 (br s, 2H), 6.72 (t, 2H, *J* = 7.7 Hz), 6.68 (d, 2H, *J* = 7.7 Hz), 6.33 (s, 2H), 6.08 (br s, 2H). ^13^C {^1^H} NMR (CDCl_3_, 150 MHz): δ 180.4, 153.1, 138.6, 133.3 (q, *C*_CF_ = 33.8 Hz), 130.4, 130.4, 130.2, 124.3, 123.2 (q, *C*_CF_ = 272.2 Hz), 122.0, 120.0, 117.2, 61.2.^19^F NMR (CDCl_3_ 564 MHz): δ −63.06. Anal. Calcd for C_32_H_22_N_4_S_2_O_2_F_12_: C, 48.86; H, 2.82; N, 7.12. Found: C, 48.82; H, 2.86; N, 7.03.

### Synthesis **TFTPA**

3,5-Bis(trifluoromethyl)phenyl isothiocyanate (0.290 g, 1.08 mmol, 2 equiv) was added to (1*R*,*2R*)-1,2-diphenylethane-1,2-diamine (0.115 g, 0.54 mmol, 1 equiv) in CH_2_Cl_2_ (20 mL) at room temperature, under a nitrogen atmosphere. The reaction mixture was stirred at room temperature for 24 h and monitored by ^1^H NMR. The solvent was removed by evaporation under vacuum to afford chemically pure **TFTPA** in a nearly quantitative yield.

**TFTPA**. White amorphous solid (0.390 g, 0.51 mmol, 94% yield), ^1^H NMR (CDCl_3_, 600 MHz): δ 7.98 (s, 2H), 7.72 (s, 4H), 7.70 (s, 2H), 7.48 (br s, 2H), 7.25–7.22 (m, 6H), 7.16 (br s, 4H), 6.01 (br s, 2H). ^13^C {^1^H} NMR (CDCl_3_, 150 MHz): δ 180.6, 138.4, 136.7, 133.1 (q, *C*_CF_ = 34.5 Hz), 129.1, 128.6, 127.6, 123.9, 122.7 (q, *C*_CF_ = 273.1 Hz), 119.7, 64.9.

## Supporting Information Available

Nonequivalences and enantioresolution quotients for **1–3**, **11–13**, **18–23** in the presence of 1 equiv of DABCO and **BTDA** or **TFTDA**; nonequivalences and enantioresolution quotients for **1–3** DABCO in the presence of **TFTDA** or **TFTMA** at different molar ratios; nonequivalences and enantioresolution quotients for **11–13**/DABCO in the presence of **TFTDA** or **TFTMA** at different molar ratios; stoichiometry determination; nonequivalences and enantioresolution quotients for **1–17** in the presence of **TFTDA** and DABCO in C_6_D_6_ and CDCl_3_; ^1^H and ^19^F NMR spectra of **1–10** in the presence of DABCO and **TFTDA** in CDCl_3_ and C_6_D_6_; ^1^H NMR spectra of **11–17** in the presence of DABCO and **TFTDA** in CDCl_3_ and C_6_D_6_; ^1^H NMR and 1D-TOCSY spectra of **2**, **4–6**, **13,** and **17** in the presence of DABCO/**TFTDA** in C_6_D_6_; nonequivalences for **11–17** in equimolar mixtures **11–17**/DABCO/**TFTDA** as a function of concentration; nonequivalences for **11**, **13–17**/DABCO (1:1) in the presence of 1 equiv of **TFTDA** at different substrate concentration; nonequivalences for **1–10** in equimolar mixtures **1–10**/DABCO/**TFTDA** as a function of the concentration; ^1^H NMR spectra of **TFTDA** at 15 and 5 mM; ^1^H NMR chemical shift data for **TFTDA** in 15 and 5 mM solutions; ^19^F NMR spectra of **1–3** and ^1^H NMR spectra of **11–13** in the presence of 1 equiv of DABCO and of 1 or 0.3 equiv of **TFTDA**; 2D ROESY map **TFTDA** at 30 mM; diffusion coefficients of pure DABCO (15 mM) in an equimolar mixture **TFTDA** and **TFTPA**; 1D ROESY of DABCO in the presence of **TFTDA**; 1D ROESY of **16** protons in equimolar mixtures (*R*)-**16** or (*S*)-**16**/DABCO/**TFTDA**; ^1^H chemical shifts and complexation shift for (*R*)-**16** and (*S*)-**16**/DABCO/**TFTDA** equimolar mixtures; ROESY maps of **TFTDA**/substrate/DABCO; association constant determination; and ^1^H and ^13^C{^1^H} NMR spectra of **TFTMA** and **TFTDA** (PDF)
