# Supplementary material for: Bis-Thiourea Chiral Sensor for the NMR Enantiodiscrimination of N-Acetyl and N-Trifluoroacetyl Amino Acid Derivatives
Source: J Org Chem. 2022 Sep 5;87(18):11968–78. doi: 10.1021/acs.joc.2c00814 (PMC9486950; doi:10.1021/acs.joc.2c00814)
Supplement: Supplementary file 1 — jo2c00814_si_001.pdf [file jo2c00814_si_001.pdf]

A bis-thiourea chiral sensor for the NMR enantiodiscrimination of *N*-acetyl and *N*-trifluoroacetyl amino acid derivatives

Alessandra Recchimurzo, Federica Balzano,* Gloria Uccello Barretta,* Luca Gherardi

Department of Chemistry and Industrial Chemistry, University of Pisa, via Moruzzi 13, 56124 Pisa, Italy.

*federica.balzano@unipi.it

*gloria.uccello.barretta@unipi.it

Supporting Information

Table S1. Nonequivalences and enantioresolution quotients for **1**-**3**, **11**-**13**, **18**-**23** in the presence of 1 equivalent of DABCO and **BTDA** or **TFTDA**.

Table S2. Nonequivalences and enantioresolution quotients for **1**-**3**/DABCO in the presence of **TFTDA** or **TFTMA** at different molar ratios.

Table S3. Nonequivalences and enantioresolution quotients for **11**-**13**/DABCO in the presence of **TFTDA** or **TFTMA** at different molar ratios.

Figure S1. Stoichiometry determination

Table S4. Nonequivalences and enantioresolution quotients for **1-17** in the presence of **TFTDA** and DABCO in C_6D_6 and $CDCl_3$.

Figure S2. ^{1}H and ^{19}F NMR spectra of **1**-**6** in the presence of DABCO and **TFTDA** in $CDCl_3$ and C_6D_6.

Figure S3. ^{1}H and ^{19}F NMR spectra of **7**-**9** in the presence of DABCO and **TFTDA** in $CDCl_3$ and C_6D_6.

Figure S4. ^{1}H NMR spectra of **11**-**17** in the presence of DABCO and **TFTDA** in $CDCl_3$ and C_6D_6.

Figure S5. ^{1}H NMR and 1D-TOCSY spectra of **2**, **4**-**6**, **13** and **17** in the presence of DABCO/**TFTDA** in C_6D_6.

Figure S6. Nonequivalences for **11**-**17** in equimolar mixtures **11**-**17**/DABCO/**TFTDA** as a function of concentration.

Table S5. Nonequivalences for **11**, **13**-**17**/DABCO (1:1) in the presence of 1 equivalent of **TFTDA** at different substrate concentration.

Figure S7. Nonequivalences for **1**-**10** in equimolar mixtures **1**-**10**/DABCO/**TFTDA** as a function of concentration.

Figure S8. ^{1}H NMR spectra of **TFTDA** at 15 mM and 5 mM.

Table S6. ^{1}H NMR chemical shift data for **TFTDA** in 15 mM and 5 mM solutions.

Figure S9. ^{19}F NMR spectra of **1**-**3** and ^{1}H NMR spectra of **11**-**13** in the presence of 1 equivalent of DABCO and 1 or 0.3 equivalents of **TFTDA**.

Figure S10. 2D ROESY map of **TFTDA** at 30 mM.

Table S7. Diffusion coefficients of pure DABCO (15 mM) an in equimolar mixture with **TFTDA** and **TFTPA**.

Figure S11. 1D ROESY spectra of DABCO in the presence of **TFTDA** and in (*S*)-**16**/DABCO/TFTDA or (*R*)-**16**/DABCO/TFTDA equimolar mixtures.

Figure S12. 1D ROESY spectra of acetyl protons of **16** in equimolar mixtures (*R*)-**16**/DABCO/**TFTDA** or (*S*)-**16**/DABCO/**TFTDA**.

Figure S13. 1D ROESY spectra of methine protons of isobutyl group of **16** in equimolar mixtures (*R*)-**16**/DABCO/**TFTDA** or (*S*)-**16**/DABCO/**TFTDA**.

Figure S14. 1D ROESY spectra of methine protons at the chiral center of **16** in equimolar mixtures (*R*)-**16**/DABCO/**TFTDA** or (*S*)-**16**/DABCO/**TFTDA**.

Table S8. ^{1}H NMR chemical shifts and complexation shifts for (*R*)-**16** and (*S*)-**16**/DABCO/**TFTDA** equimolar mixtures (15 mM).

Figure S15. 2D ROESY maps of mixtures (*R*)- or (*S*)-**16**/**TFTDA**/DABCO and , c) (*R*)- or (*S*)-**13**/**TFTDA**/DABCO

Figure S16. 2D ROESY maps of mixtures (*R*)- or (*S*)-**3**/**TFTDA**/DABCO, (*RS*)-**2**/**TFTDA**/DABCO, and (*RS*)-**5**/**TFTDA**/DABCO

Figure S17. Association constants determination

Figure S18-S19. ^{1}H and ^{13}C{^{1}H} NMR spectra of **TFTMA**.

Figure S20-S21. ^{1}H and ^{13}C{^{1}H} NMR spectra of **TFTDA**.

Table S1. ^{1}H (600 MHz) and ^{19}F (564 MHz) nonequivalences ($\Delta\Delta\delta=|\delta_R-\delta_S|$, ppm; $CDCl_3$, 25 °C) and enantioresolution quotients (E, in parentheses) for **1**-**3**, **11**-**13**, **18**-**23** (15 mM) in the presence of 1 equivalent of DABCO and **BTDA** or **TFTDA**

	**BTDA**					**TFTDA**				
sub	NH	CH	oDNB<br>pDNB	Ac/OMe	CF_3	NH	CH	oDNB<br>pDNB	Ac/OMe	CF_3
**1**	0.022<br>(0.3)	0.012<br>(0.2)			0.021<br>(1.1)	0.054<br>(0.9)	0.012<br>(0.2)			0.066<br>(8.1)
**2**	0.012<br>(0.2)	0.013<br>(0.2)			0.006<br>(0.3)	0.048<br>(0.8)	0.021<br>(0.5)			0.099<br>(13.6)
**3**	0.033<br>(0.6)	0.021<br>(0.4)			0.008<br>(0.4)	0.082<br>(1.5)	0.096<br>(1.8)			0.050<br>(7.0)
**11**	0.089<br>(1.7)	0.030<br>(0.3)		0.093<br>(15.5)		0.152<br>(2.3)	0.012<br>(0.2)		0.196<br>(28.0)	
**12**	0.035<br>(0.6)	0.032<br>(0.4)		0.075<br>(12.5)		0.066<br>(1.0)	0.036<br>(0.6)		0.129<br>(18.4)	
**13**	0.040<br>(0.7)	0.016<br>(0.4)		0.095<br>(15.8)		0.130<br>(2.0)	0.106<br>(2.1)		0.092<br>(13.3)	
**18**	0.093<br>(2.1)	0.038<br>(0.5)	0.187 (12.6)<br>0.128 (5.8)			0.199<br>(3.3)	0.058<br>(0.4)	0.026 (2.5)<br>0.002 (0.1)		
**19**	0.020<br>(0.4)	0.086<br>(1.2)	0.175 (11.7)<br>0.124 (5.6)			0.087<br>(1.5)	0.008<br>(0.1)	0.014 (1.3)<br>0.013 (0.7)		
**20**	0.146<br>(3.2)	0.055<br>(0.9)	0.208 (13.9)<br>0.185 (8.4)			—	0.048<br>(0.8)	0.002 (0.2)<br>0.013 (0.7)		
**21**	—	0.005<br>(0.1)	0.091 (6.6)<br>0.066 (3.7)	0.007<br>(1.2)		—	0.012<br>(0.3)	0.016 (1.5)<br>0.023 (1.2)	0.004<br>(0.7)	
**22**	0.018<br>(0.4)	0.003<br>(0.1)	0.051 (3.4)<br>0.036 (2.0)	0.006<br>(1.0)		—	0.002<br>(0)	0.008 (0.8)<br>—	—	
**23**	0.221<br>(4.9)	0.030<br>(0.8)	0.134 (8.9)<br>0.112 (6.2)	0.026<br>(4.3)		0.014<br>(0.3)	0.017<br>(0.4)	0.014 (1.1)<br>0.011 (0.7)	—	

Table S2. ^{1}H (600 MHz) and ^{19}F (564 MHz) nonequivalences ($\Delta\Delta\delta=|\delta_R-\delta_S|$, ppm; $CDCl_3$, 25 °C) and enantioresolution quotients (E in parentheses) for **1**-**3** (15 mM)/DABCO (1:1) in the presence of **TFTDA** or **TFTMA** at different molar ratios

	**ΔΔδ (ppm)**						
CSA/sub	**TFTDA/1**	**TFTMA/1**	**TFTDA/2**	**TFTMA/2**	**TFTDA/3**	**TFTMA/3**	
1:1	0.054<br>(0.9)	0.001<br>(0.01)	0.048<br>(0.8)	0.038<br>(0.5)	0.082<br>(1.5)	—	NH
	0.066<br>(8.1)	0.018<br>(1.9)	0.099<br>(13.6)	0.004<br>(0.1)	0.050<br>(7.0)	0.014<br>(1.5)	CF_3
2:1		0.053<br>(0.7)		0.043<br>(0.6)		0.035<br>(0.5)	NH
		0.023<br>(2.4)		0.007<br>(0.4)		0.023<br>(2.4)	CF_3

Table S3. ^{1}H nonequivalences (600 MHz, $\Delta\Delta\delta=|\delta_R-\delta_S|$, ppm; $CDCl_3$, 25 °C) and enantioresolution quotients (E in parentheses) for **11**-**13** (15 mM)/DABCO (1:1) in the presence of **TFTDA** or **TFTMA** at different molar ratios

	ΔΔδ (ppm)						
CSA/sub	**TFTDA/11**	**TFTMA/11**	**TFTDA/12**	**TFTMA/12**	**TFTDA/13**	**TFTMA/13**	
1:1	0.152 (2.3)	0.042 (0.7)	0.066 (1.0)	0.008 (0.1)	0.130 (2.0)	0.072 (1.2)	NH
	0.196 (28.0)	0.104 (11.6)	0.129 (18.4)	0.080 (8.9)	0.092 (13.3)	0.059 (6.5)	Ac
2:1		0.053 (1.2)		0.006 (0.1)		0.022 (0.5)	NH
		0.119 (14.0)		0.099 (11.6)		0.084 (9.8)	Ac

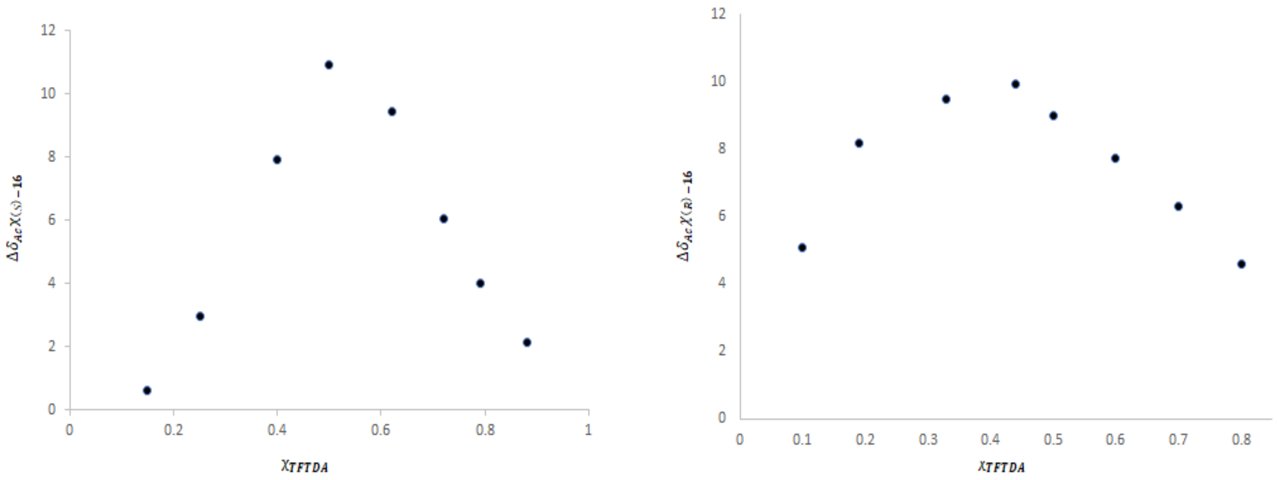

Figure S1. Stoichiometry determination based on Ac group for (*S*)-**16**/DABCO/**TFTDA** (left) and (*R*)-**16**/DABCO/**TFTDA** (right) complexes.

Table S4. ^1H (600 MHz) and ^{19}F (564 MHz) nonequivalences ($\Delta\Delta\delta=|\delta_R-\delta_S|$, ppm, 25 °C) and enantioresolution quotients (E in parentheses) for **1-17** (15 mM) in equimolar mixtures with **TFTDA** and in the presence of 1 equivalent (for **1**-**9** and **11**-**17**) or 2 equivalents (for **10**) of DABCO in C_6D_6 and $CDCl_3$

	**ΔΔδ (ppm)**			
	**C_6D_6**		**$CDCl_3$**	
sub	**NH**	**CF_3/Ac**	**NH**	**CF_3/Ac**
**1**	0.089 (0.9)	0.026 (1.7)	0.054 (0.9)	0.066 (8.1)
**2**	—	0.007 (0.5)	0.048 (0.8)	0.099 (13.6)
**3**	—	0.008 (0.5)	0.082 (1.5)	0.050 (7.0)
**4**	—	0.113 (7.5)	0.222 (3.9)	0.093 (13.5)
**5**	0.092 (0.9)	0.012 (0.8)	—	0.087 (12.6)
**6**	0.070 (0.8)	—	0.110 (1.9)	0.090 (9.7)
**7**	0.008 (0.1)	0.017 (1.1)	—	0.078 (11.2)
**8**		0.094 (6.3)/0.130 (8.7)		0.042 (6.1)/0.034 (4.9)
**9**	0.105 (1.1)	0.016 (1.1)	—	0.025 (3.6)
**10**[a]			1.249 (21.9)	0.219 (31.3)
**11**	0.287 (4.2)	0.193 (10.7)	0.152 (2.3)	0.196 (28.0)
**12**	0.055 (0.8)	0.070 (3.9)	0.066 (1.0)	0.129 (18.4)
**13**	0.217 (3.1)	0.062 (3.4)	0.130 (2.0)	0.092 (13.3)
**14**	0.285 (4.1)	0.137 (7.6)	0.166 (2.5)	0.171 (24.4)
**15**	0.209 (3.0)	0.117 (6.5)	0.112 (1.7)	0.144 (20.6)
**16**	0.346 (5.0)	0.195 (10.8)	0.156 (2.4)	0.191 (27.3)
**17**	0.170 (2.5)	0.133 (7.2)	0.057 (0.9)	0.171 (24.4)

[a]**10** is not soluble in C_6D_6 even in the presence of 2 equivalents of DABCO and 1 equivalent of CSA.

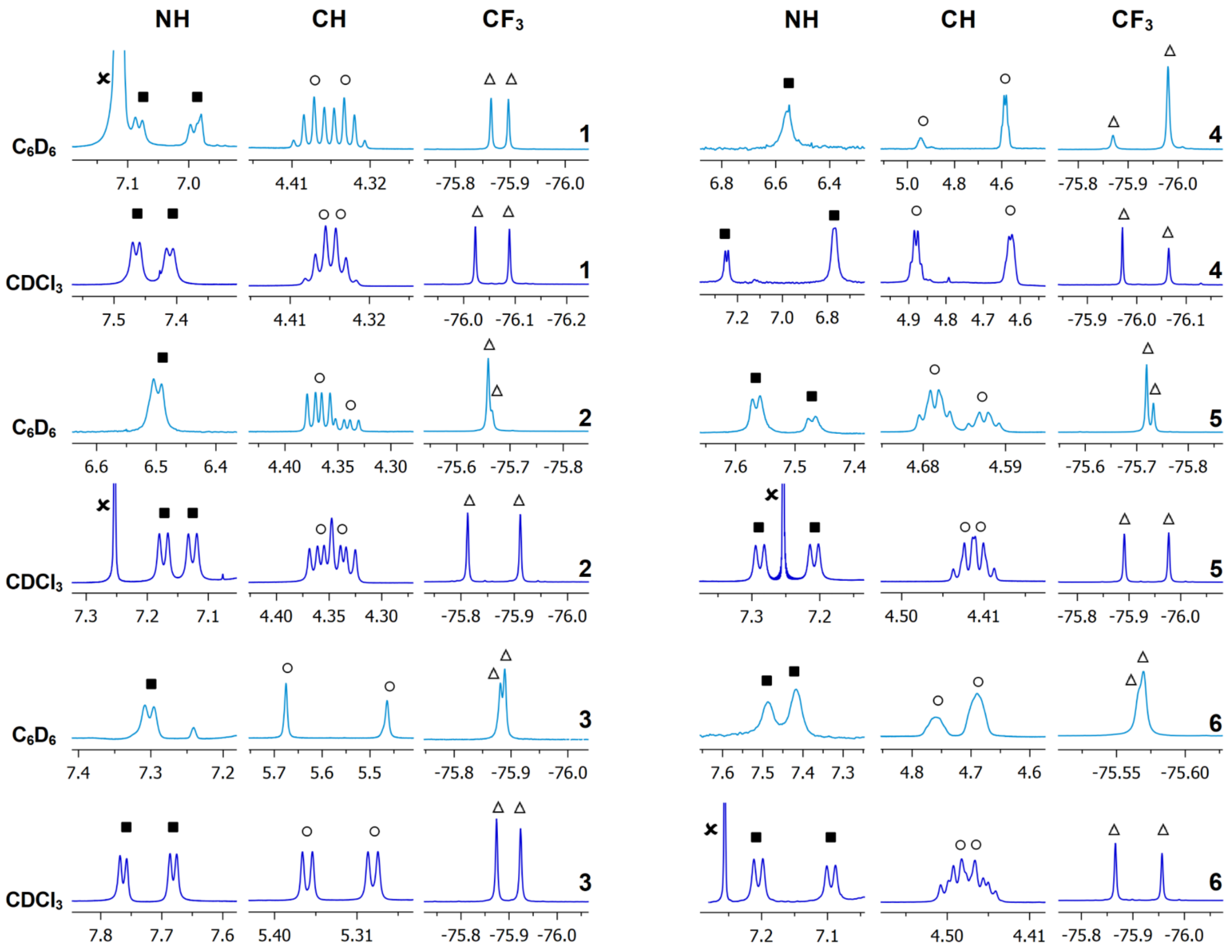

Figure S2. ^{19}F NMR (564 MHz, 25 °C) spectral regions corresponding to CF_3 (△) resonances of **1**-**6** (15 mM) and ^{1}H NMR (600 MHz, 25 °C) spectral regions corresponding to NH (■) and CH-α (○) resonances of **1**-**6** (15 mM) in the presence of 1 equivalent of DABCO and **TFTDA** in $CDCl_3$ and C_6D_6. × indicates solvent resonances. NH resonances of **3** and **4** in $CDCl_3$ and of **2**, **4**-**6** in C_6D_6 were extracted by 1D TOCSY experiments carried out by selective perturbation of CH-α protons: corresponding 1D TOCSY spectra are shown in the figure. Racemic or enantiomerically enriched samples of amino acid derivatives were analyzed.

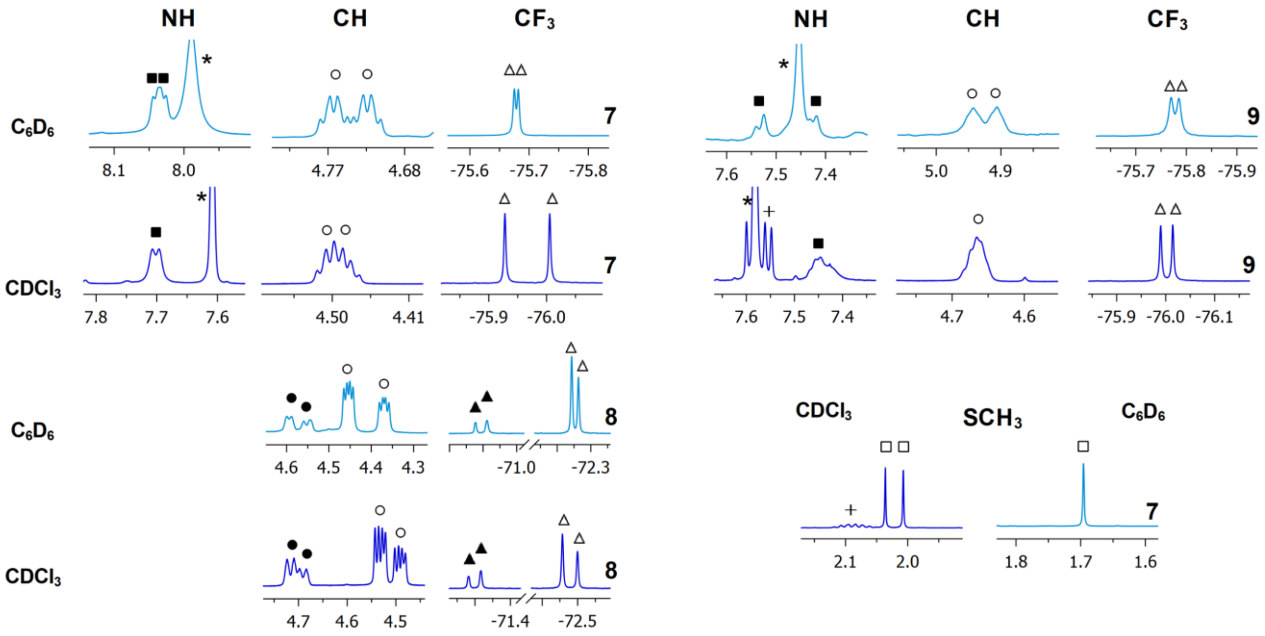

Figure S3. ^{19}F NMR (564 MHz, 25 °C) spectral regions corresponding to CF_3 (△) resonances of **7**-**9** (15 mM) and ^{1}H NMR (600 MHz, 25 °C) spectral regions corresponding to NH (■) and CH-α (○) resonances of **7**-**9** (15 mM) and to methylthio (□) resonances of **7** in the presence of 1 equivalent of DABCO and **TFTDA** in $CDCl_3$ and C_6D_6. For **8**, ● and ▲ indicate the resonances of the *syn* stereoisomer. + and * indicate side chain protons of amino acid and CSA resonances, respectively. Racemic or enantiomerically enriched samples of amino acid derivatives were analyzed.

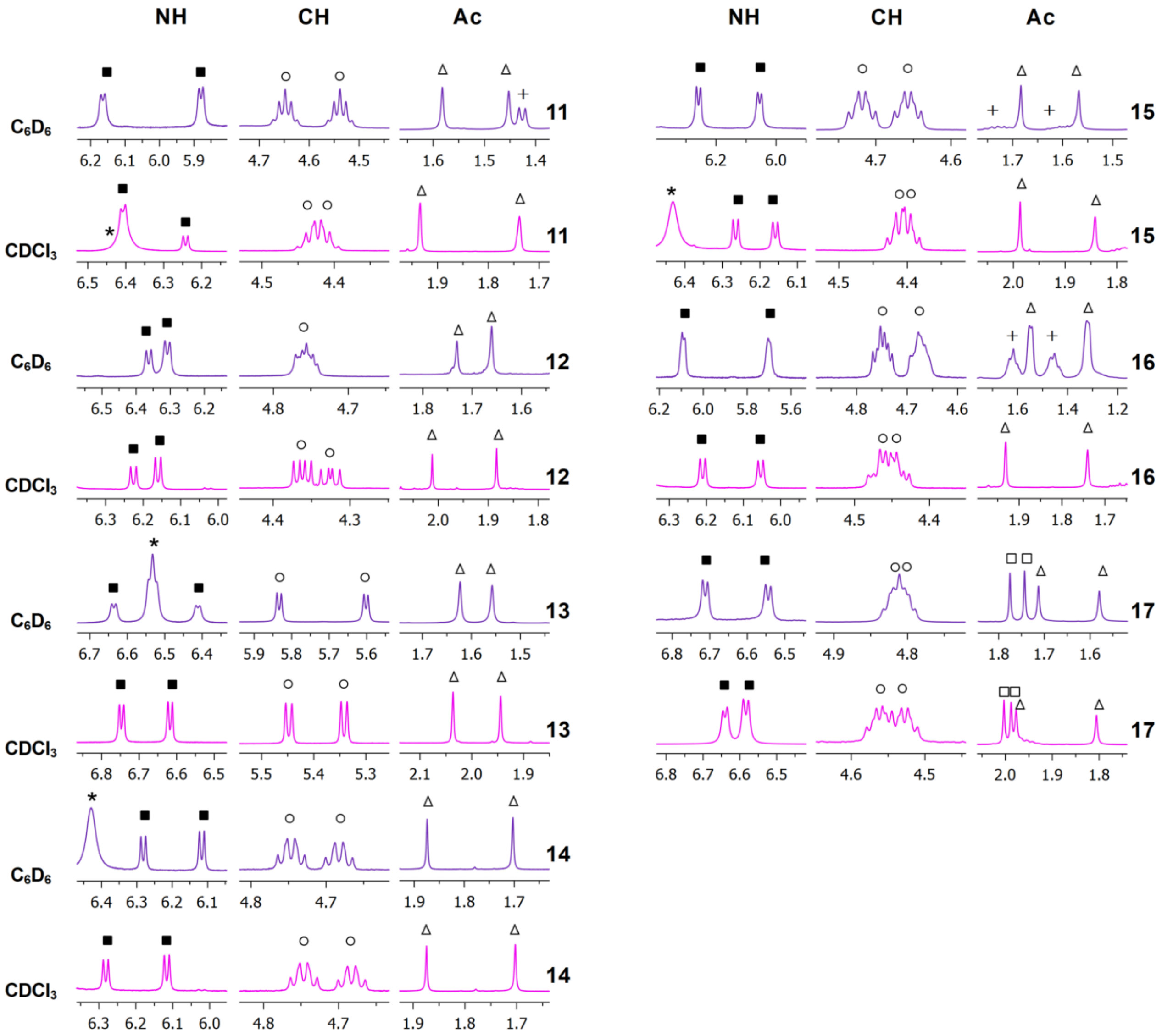

Figure S4. ^{1}H NMR (600 MHz, 25 °C) spectral regions corresponding to NH (■), CH-α (○), acetyl (△) resonances of **11**-**17** (15 mM) and to methylthio (□) resonances of **17** in the presence of 1 equivalent of DABCO and **TFTDA** in $CDCl_3$ and C_6D_6. + and * indicate side chain protons of amino acid and CSA resonances, respectively. NH resonances of **13** and **17** in $CDCl_3$ and in C_6D_6 were extracted by 1D TOCSY experiments carried out by selective perturbation of CH-α protons: corresponding 1D TOCSY spectra are shown in the figure. Racemic or enantiomerically enriched samples of amino acid derivatives were analyzed.

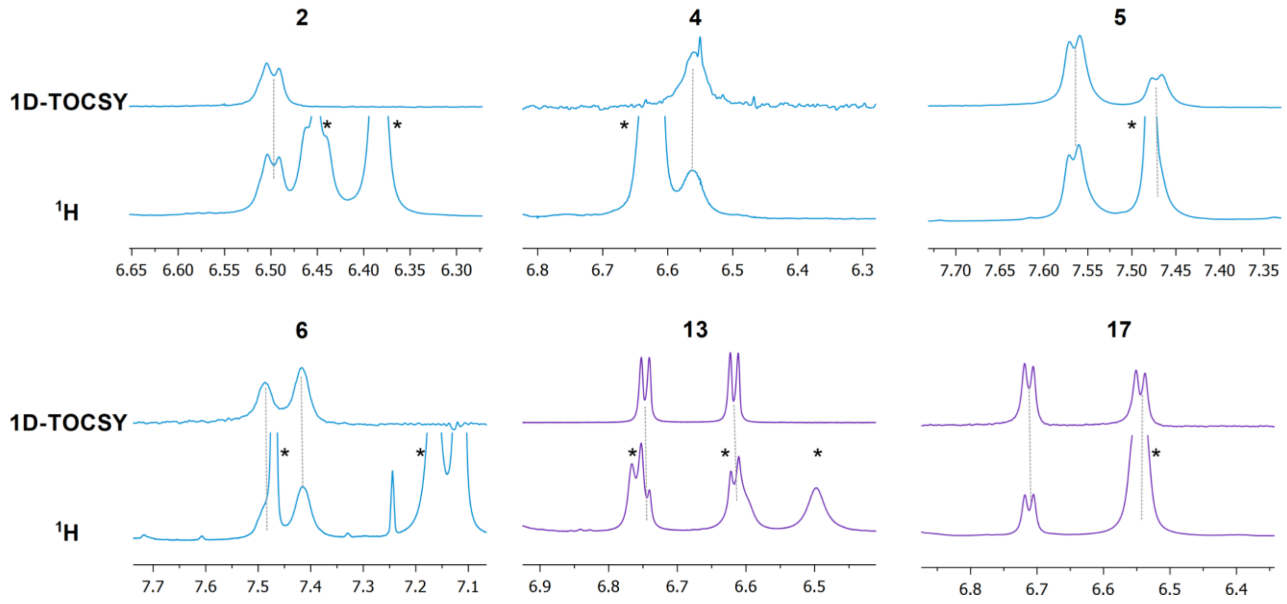

Figure S5. ^{1}H NMR (600 MHz, 25 °C, C_6D_6) spectrum (bottom) and 1D-TOCSY experiments (top) obtained by selective excitation of methine proton of **2**, **4**-**6**, **13** and **17** (15 mM) in the presence of 1 equivalent of DABCO/**TFTDA**.
* indicates CSA resonances. Racemic or enantiomerically enriched samples of amino acid derivatives were analyzed.

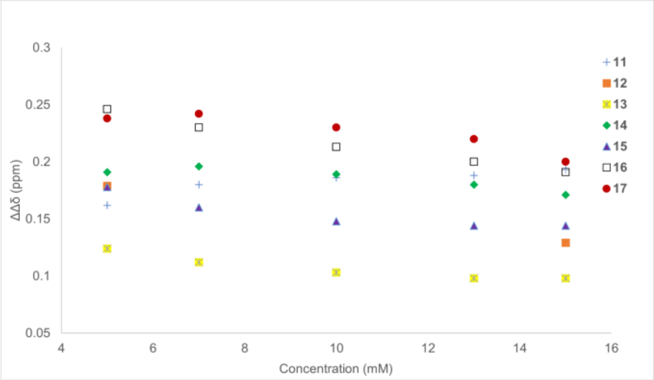

Figure S6. ^{1}H nonequivalences (600 MHz, $\Delta\Delta\delta=|\delta_R-\delta_S|$; ppm $CDCl_3$, 25 °C) of acetyl protons for **11-17** in equimolar mixtures **11-17**/DABCO/**TFTDA** as a function of concentration

Table S5. ^{1}H nonequivalences (600 MHz, $\Delta\Delta\delta=|\delta_R-\delta_S|$, ppm; $CDCl_3$, 25 °C) for **11**, **13-17**/DABCO (1:1) in the presence of 1 equivalent of **TFTDA** at different substrate concentration

		ΔΔδ (ppm)				
sub		**15 mM**	**13 mM**	**10 mM**	**7 mM**	**5 mM**
**11**	NH	0.152	0.142	0.139	0.125	0.108
	CH	—	—	—	—	—
	Ac	0.196	0.188	0.186	0.180	0.163
**13**	NH	0.130	0.128	0.128	0.130	0.129
	CH	0.107	0.105	0.105	0.102	0.099
	Ac	0.092	0.098	0.103	0.112	0.117
**14**	NH	0.166	0.167	0.167	0.165	0.158
	CH	0.063	0.065	0.068	0.071	0.067
	Ac	0.171	0.180	0.189	0.196	0.191
**15**	NH	0.112	0.109	0.110	0.112	0.118
	CH	—	—	—	—	—
	Ac	0.144	0.144	0.148	0.160	0.178
**16**	NH	0.156	0.157	0.160	0.160	0.157
	CH	—	—	—	—	—
	Ac	0.191	0.200	0.213	0.230	0.246
**17**	NH	0.057	0.036	0.033	0.025	—
	CH	0.044	0.049	0.053	0.057	0.053
	Ac	0.171	0.220	0.230	0.242	0.238
	SMe	0.021	0.024	0.033	0.040	0.051

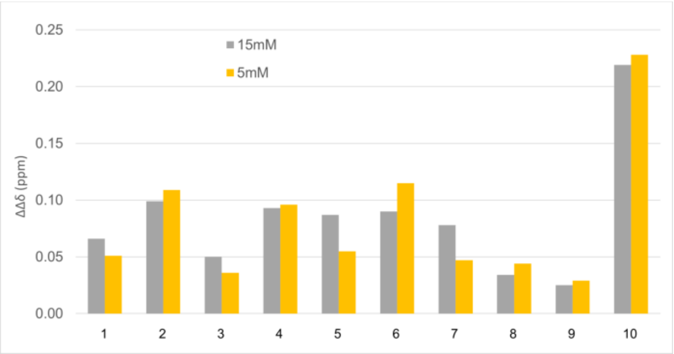

Figure S7. ^{1}H nonequivalences (600 MHz, $\Delta\Delta\delta=|\delta_R-\delta_S|$; ppm $CDCl_3$, 25 °C) of CF_3 for **1-10** (15 mM in grey and 5 mM in yellow) in equimolar mixtures **1-10**/DABCO/**TFTDA**

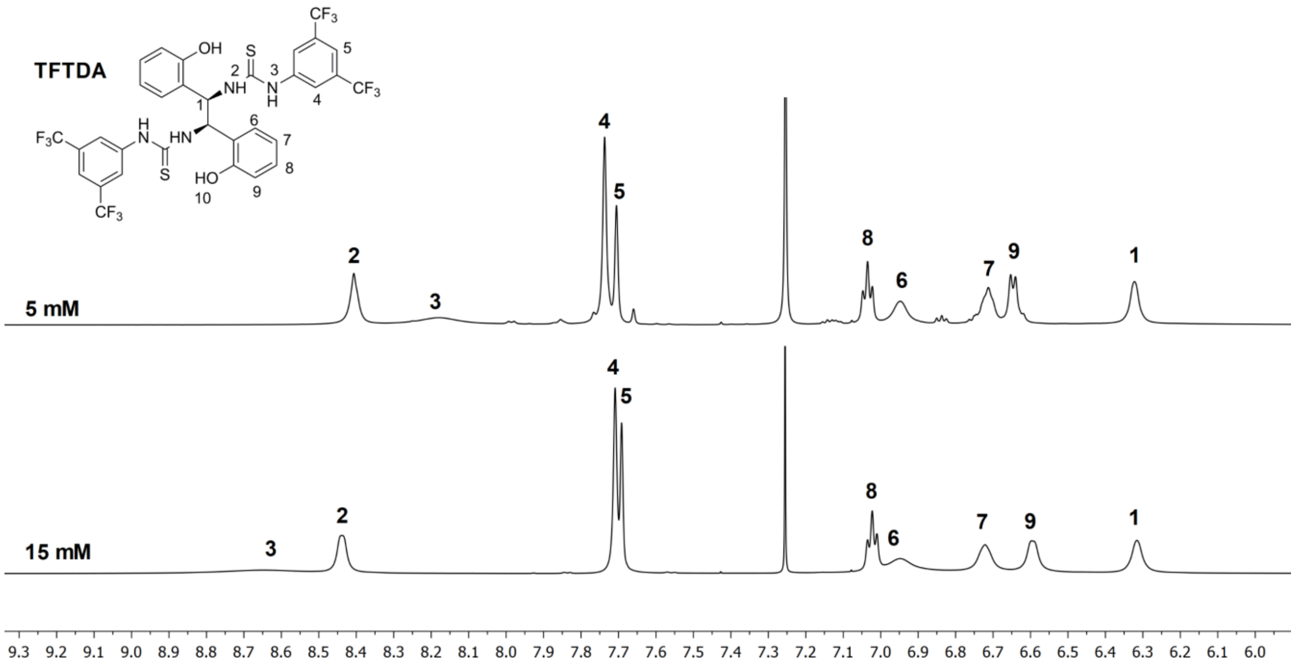

Figure S8. ^{1}H NMR (600 MHz, $CDCl_3$, 25 °C) spectra of **TFTDA** (15 mM and 5 mM).

Table S6. ^{1}H NMR (600 MHz, $CDCl_3$, 25 °C) chemical shift (δ, ppm) data for **TFTDA** in 15 mM and 5 mM solutions

	**δ (ppm)**		**$\Delta\delta = \delta_{15mM} - \delta_{5mM}$**
	**15 mM**	**5 mM**	
CH(1)	6.31	6.27	0.04
NH(2)	8.44	8.41	0.03
NH(3)	8.64	8.18	0.46
H(4)	7.70	7.73	-0.03
H(5)	7.69	7.70	-0.01
H(6)	6.94	6.95	-0.01
H(7)	6.72	6.71	0.01
H(8)	7.02	7.04	-0.02
H(9)	6.59	6.64	-0.05

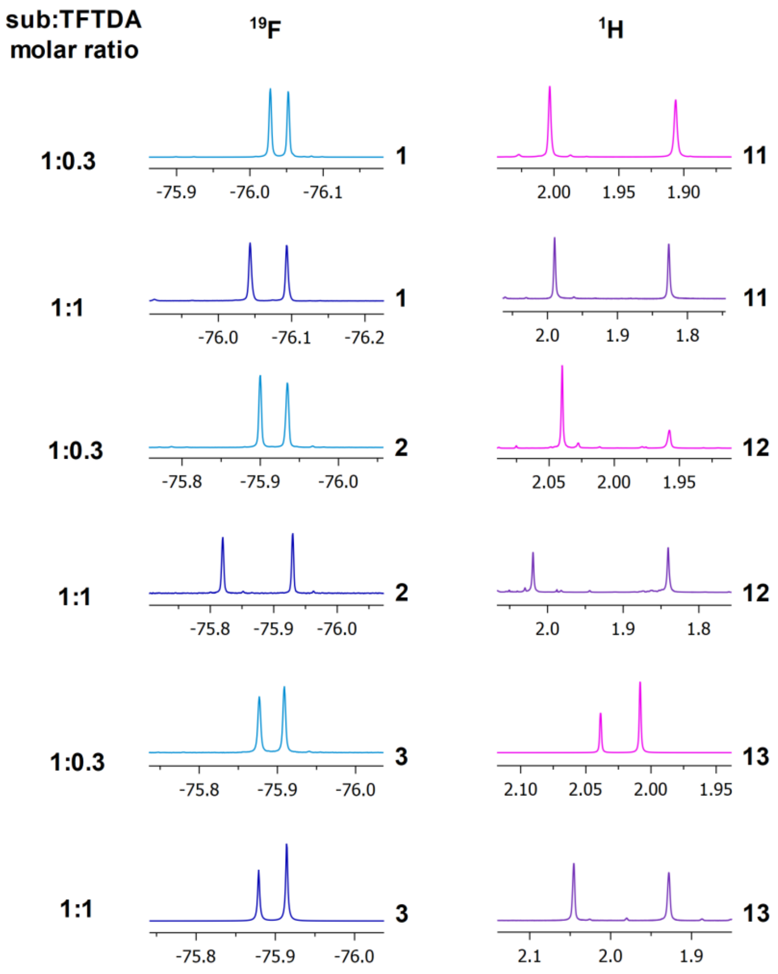

Figure S9. ^{19}F NMR (564 MHz, $CDCl_3$, 25 °C) spectral regions corresponding to CF_3 resonances of **1**-**3** (5 mM) and ^{1}H NMR (600 MHz, $CDCl_3$, 25 °C) spectral regions corresponding to acetyl resonances of **11**-**13** (5 mM) in the presence of 1 equivalent of DABCO and 1 or 0.3 equivalents of **TFTDA**. Racemic or enantiomerically enriched samples of amino acid derivatives were analyzed.

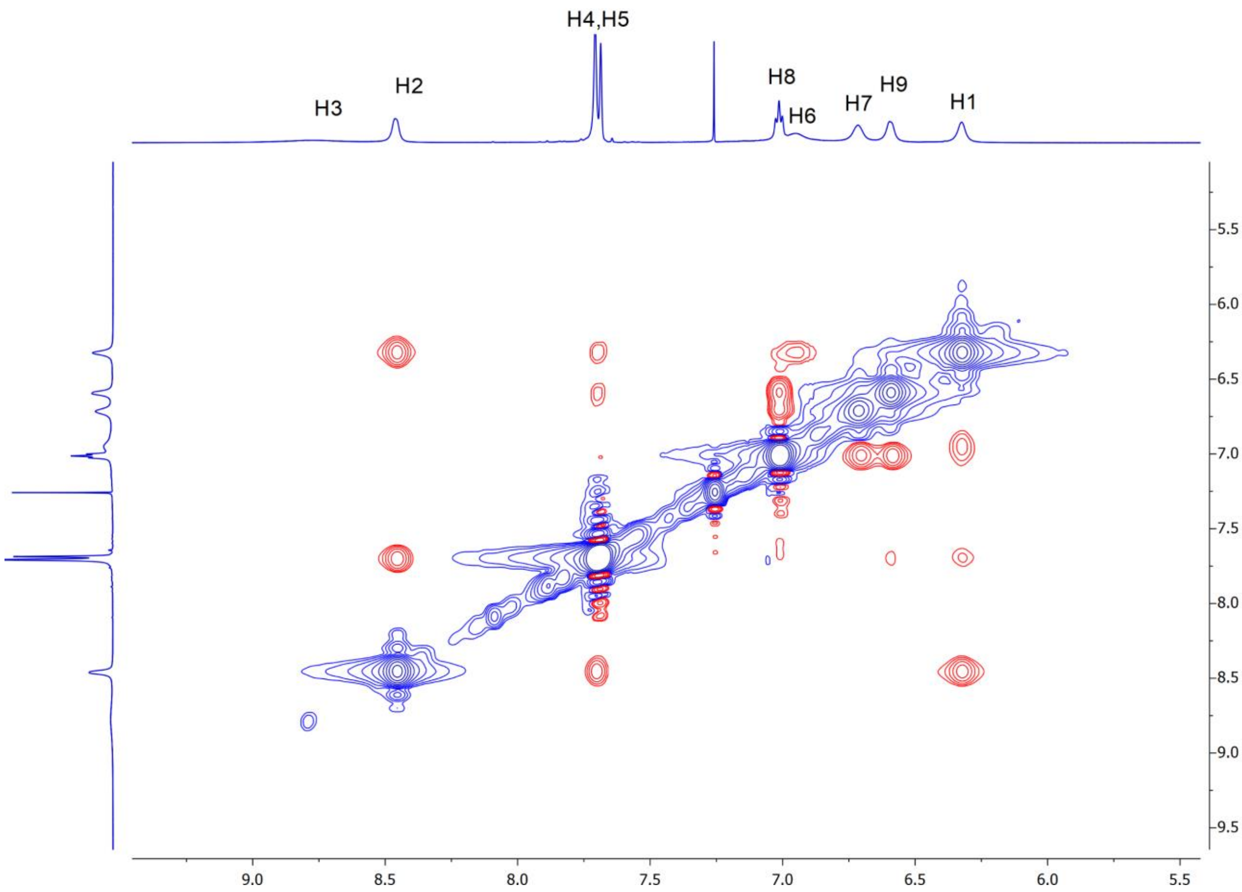

Figure S10. 2D ROESY (600 MHz, $CDCl_3$, 25 °C, mixing in time 0.3 s) map of **TFTDA** (30 mM)

Table S7. ^1H NMR (600 MHz, $CDCl_3$, 25 °C) diffusion coefficient (D x 10^{10} m^2/s) of pure DABCO (15 mM) and its equimolar mixtures with **TFTDA** and **TFTPA**

	**D x 10^{10} (m^2/s)**
DABCO	14.51 ± 0.04
DABCO/**TFTDA**	7.07 ± 0.05
DABCO/**TFTPA**	13.92 ± 0.09

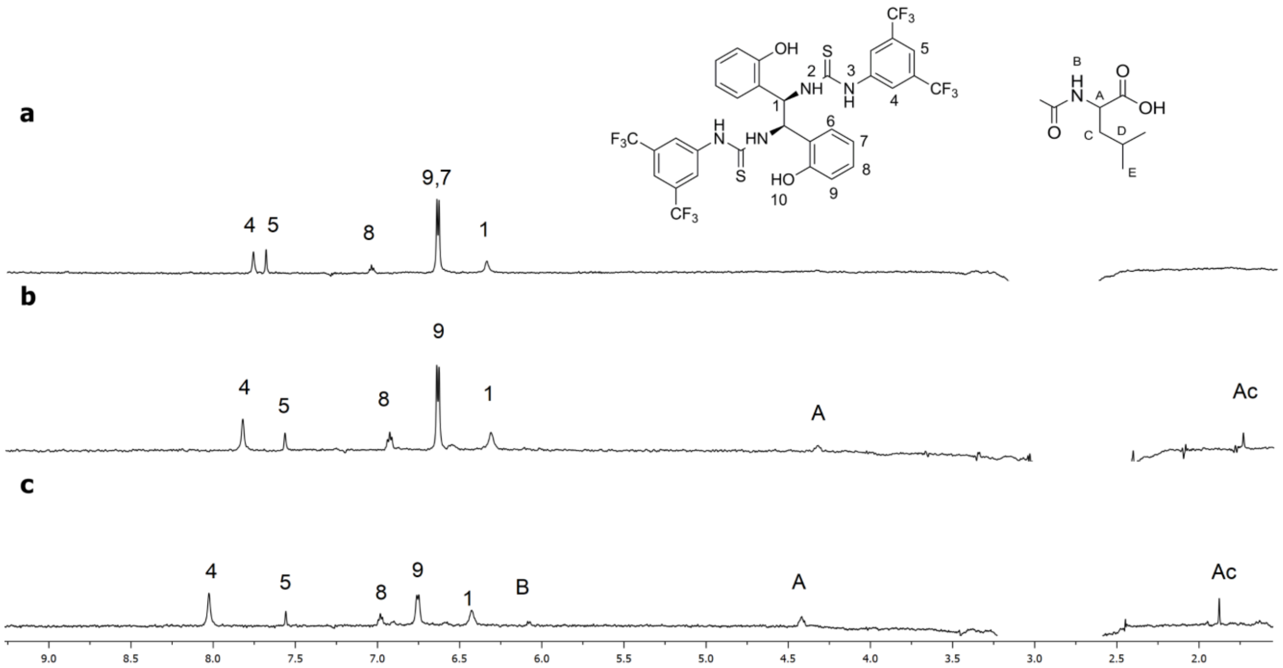

Figure S11. 1D ROESY spectra of DABCO (15 mM) in: DABCO/**TFTDA** (a), (*R*)-**16**/DABCO/**TFTDA** (b) and (*S*)-**16**/DABCO/**TFTDA** (c) equimolar mixture.

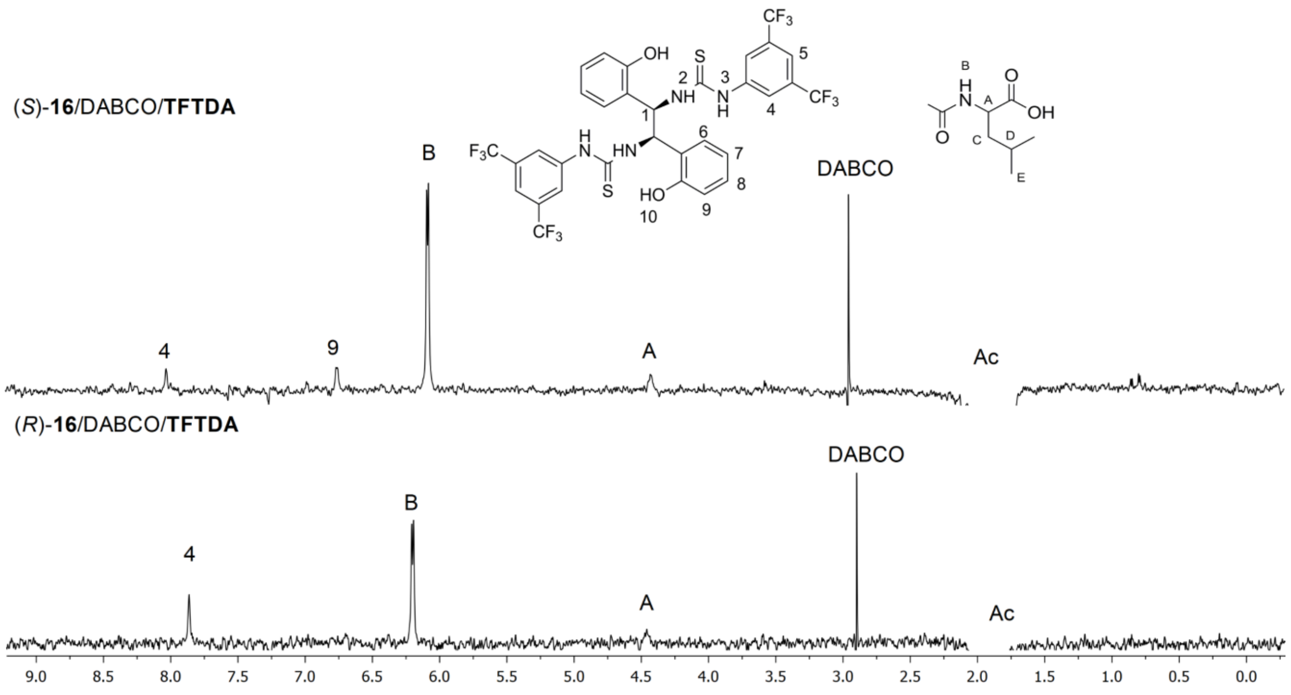

Figure S12. Comparison of 1D ROESY spectra of acetyl protons of **16** (15 mM) in (*S*)-**16**/DABCO/**TFTDA** and (*R*)-**16**/DABCO/**TFTDA** mixtures.

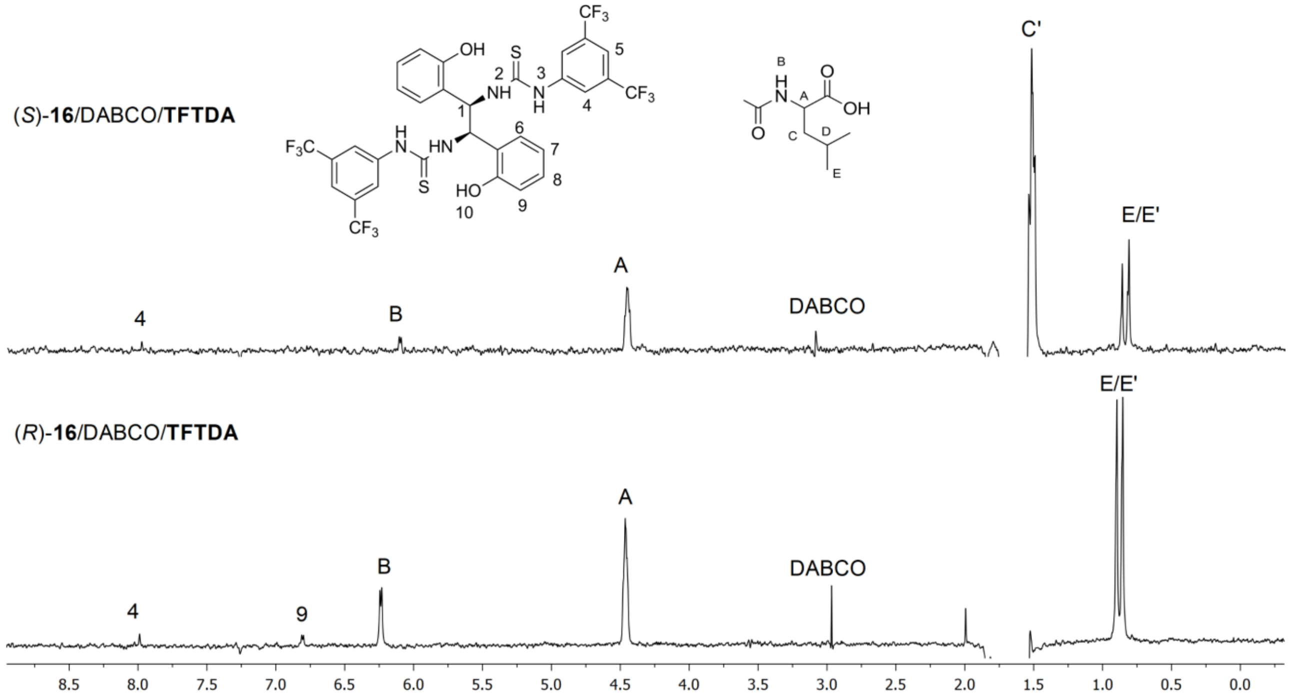

Figure S13. Comparison of 1D ROESY spectra of CH protons of isobutyl moiety of **16** (15 mM) in (*S*)-**16**/DABCO/**TFTDA** and (*R*)-**16**/DABCO/**TFTDA** mixtures.

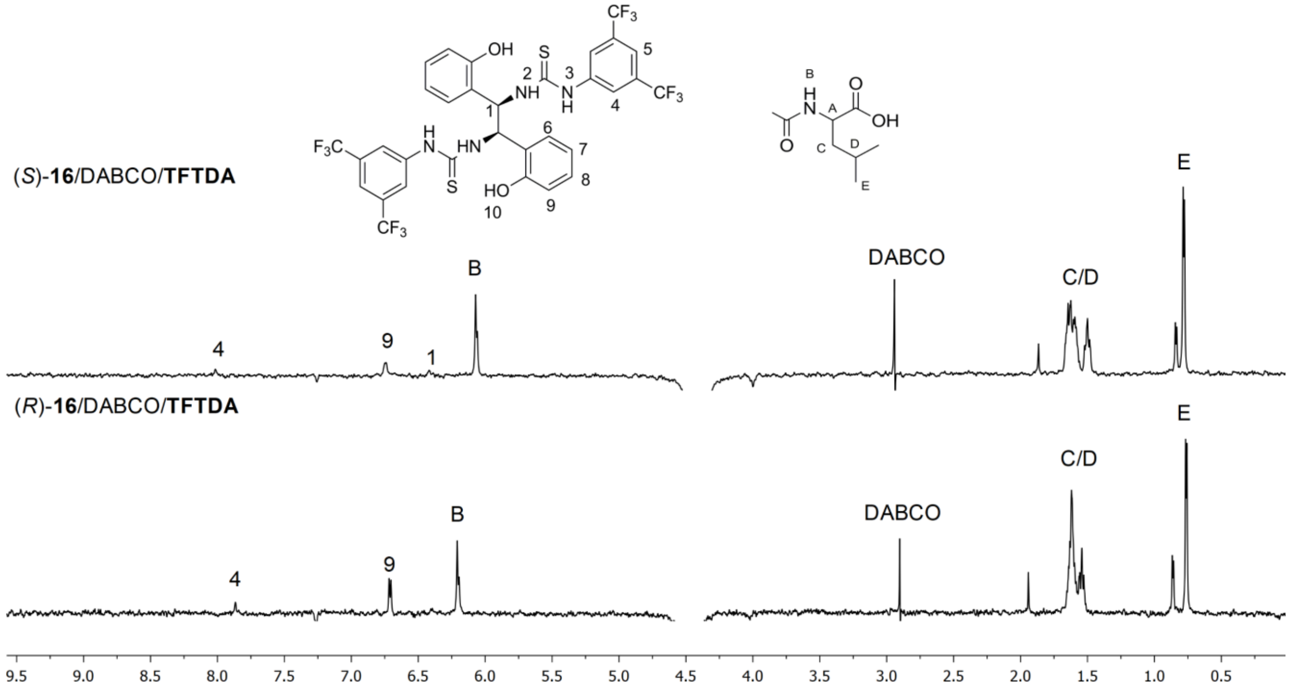

Figure S14. Comparison of 1D ROESY spectra of methine proton at the chiral center (CHα) of **16** (15 mM) in (*S*)-**16**/DABCO/**TFTDA** and (*R*)-**16**/DABCO/**TFTDA** mixtures.

Table S8. ^{1}H NMR (600 MHz, $CDCl_3$, 25 °C) chemical shifts (δ, ppm) and complexation shifts ($\Delta\delta = \delta_{mix} - \delta_f$, ppm) for (*S*)-**16** and (*R*)-**16**/**TFTDA**/DABCO equimolar mixtures (15 mM).

Protons	(*S*)-**16**/**TFTDA**/DABCO			(*R*)-**16**/**TFTDA**/DABCO	
	δ_f	δ_{mix}	Δδ	δ_{mix}	Δδ
A	4.43	4.44	0.01	4.46	0.03
B	6.12	6.04	-0.08	6.18	0.06
C	1.68	1.67	-0.01	1.62	-0.06
D	1.52	1.59	0.07	1.54	0.02
E	0.93	0.82	-0.11	0.82	-0.11
Ac	1.99	1.78	-0.21	1.94	-0.05
DABCO	2.99	3.08	0.09	2.95	-0.04
CH(1)	6.28	6.37	0.09	6.43	0.15
NH(2)	8.68	8.77	0.09	8.73	0.05
NH(3)	8.68	9.50	0.82	9.27	0.59
H(4)	7.71	7.97	0.26	7.92	0.21
H(5)	7.65	7.58	-0.07	7.59	-0.06
H(6)	6.87	7.01	0.14	7.01	0.14
H(7)	6.64	6.66	0.02	6.65	0.01
H(8)	7.01	6.99	-0.02	6.99	-0.02
H(9)	6.61	6.78	0.17	6.78	0.17

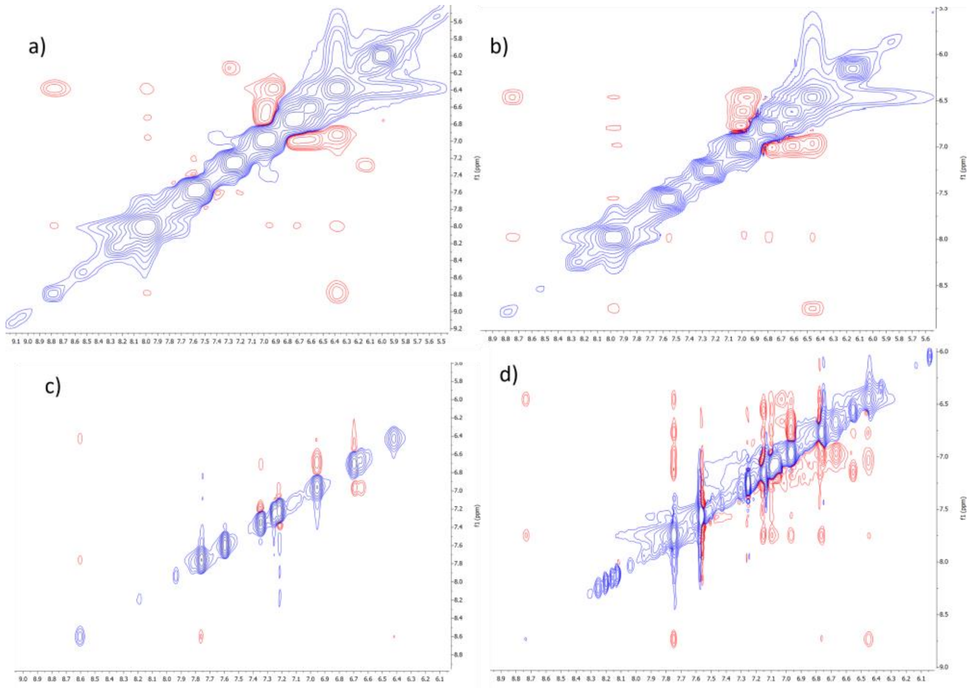

Figure S15. 2D ROESY (600 MHz, $CDCl_3$, 25 °C, mixing in time 0.3 s, 15 mM) maps of a) (*R*)-**16**/**TFTDA**/DABCO, b) (*S*)-**16**/**TFTDA**/DABCO, c) (*R*)-**13**/**TFTDA**/DABCO, d) (*S*)-**13**/**TFTDA**/DABCO

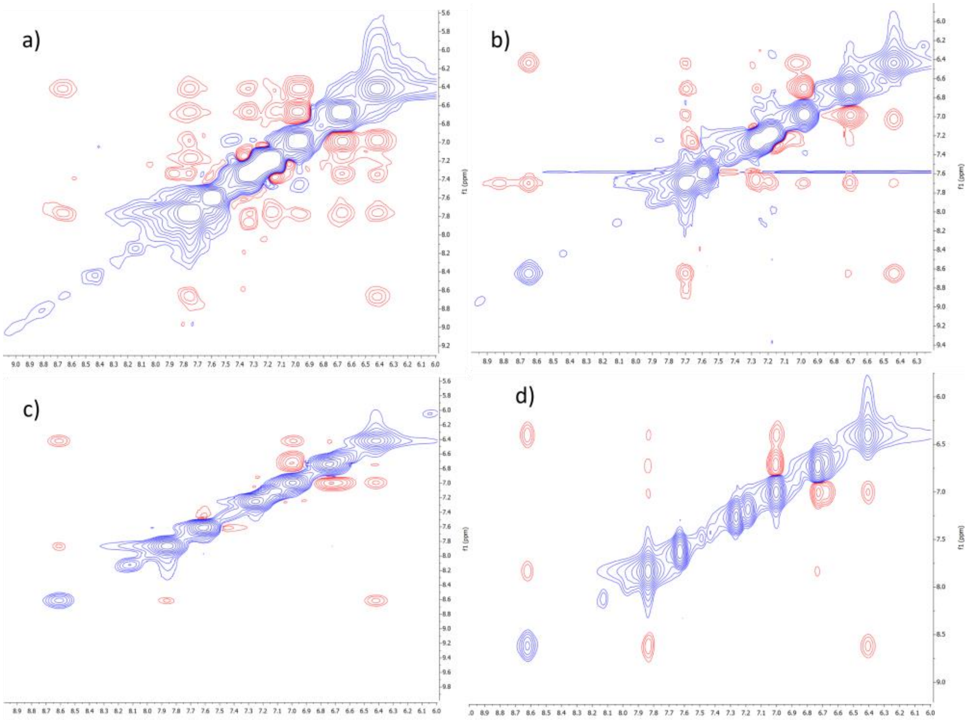

Figure S16. 2D ROESY (600 MHz, $CDCl_3$, 25 °C, mixing in time 0.3 s, 15 mM) maps of a) (*R*)-**3**/**TFTDA**/DABCO, b) (*S*)-**3**/**TFTDA**/DABCO, c) (*RS*)-**2**/**TFTDA**/DABCO, d) (*RS*)-**5**/**TFTDA**/DABCO

$$C_0 = \frac{(\delta_{obs} - \delta_f)(\delta_b - \delta_f)}{K(\delta_b - \delta_{obs})^2}$$

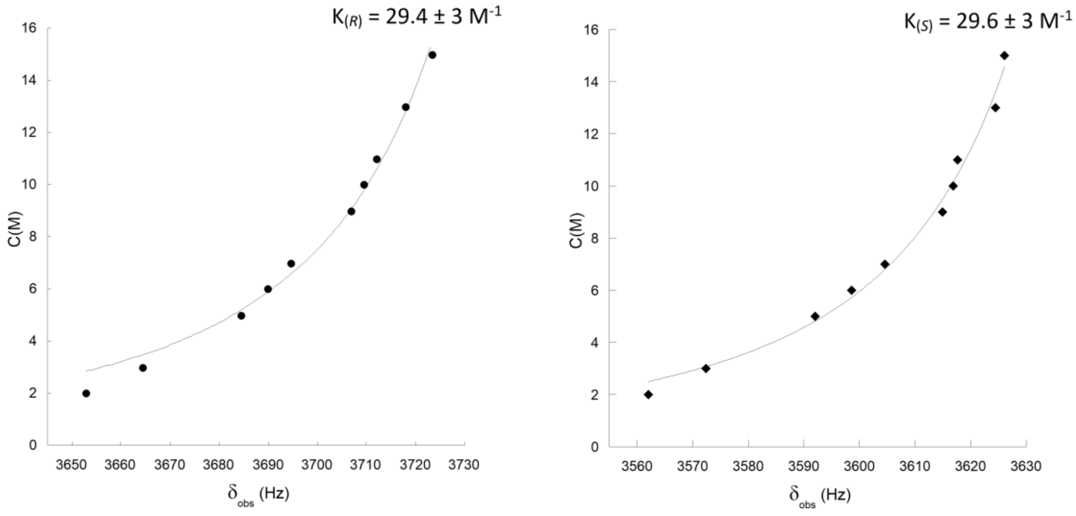

Figure S17. Non-linear fitting of dilution data: dependence of NH proton chemical shift (δ_{obs}, Hz) of **16** on the concentration (C) in equimolar mixtures (*R*)-**16**/DABCO/**TFTDA** (left) and (*S*)-**16**/DABCO/**TFTDA** (right): δ_b and δ_f in equation are the chemical shift in the bound and in the free state, respectively.

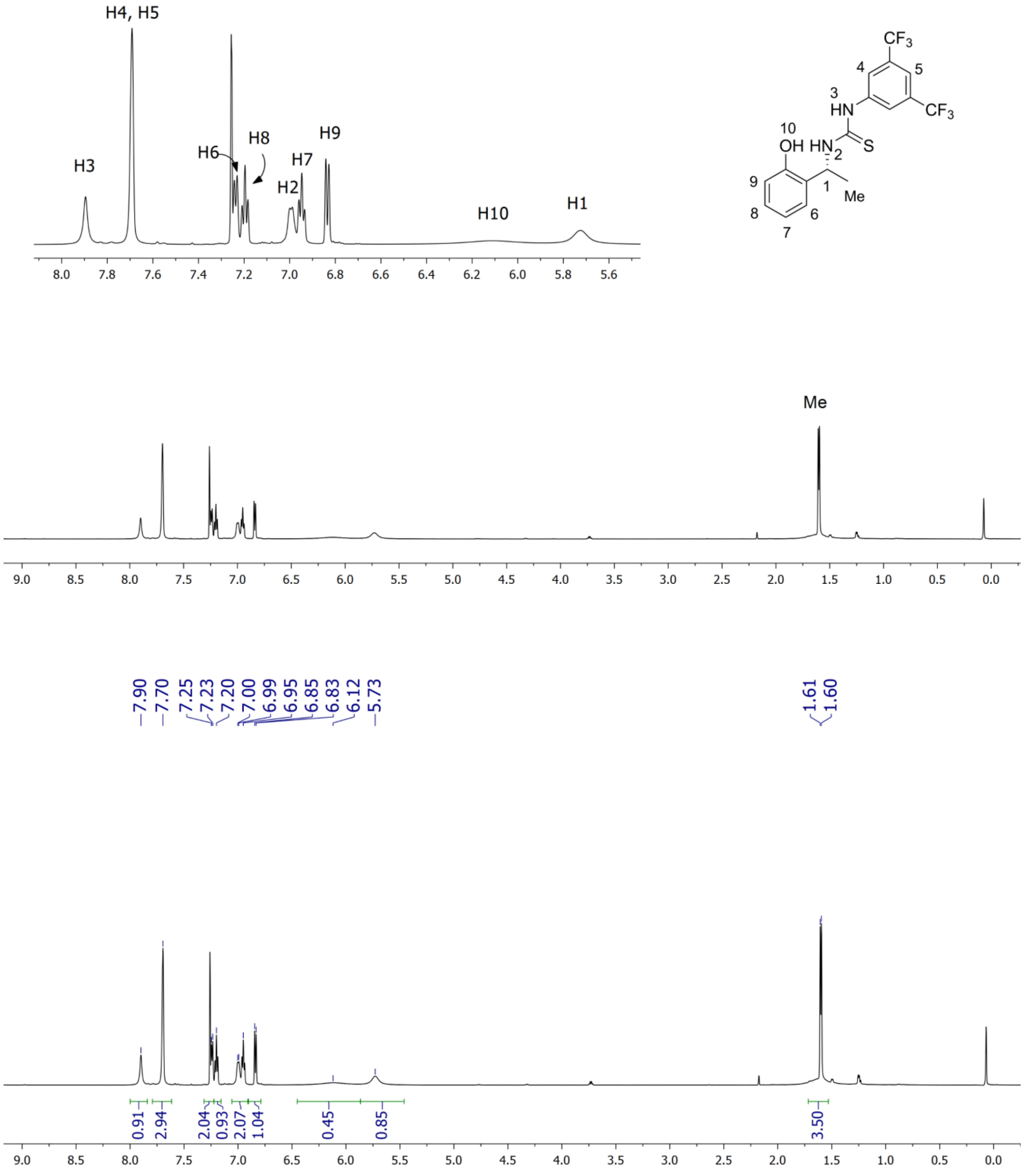

Figure S18. ^{1}H NMR (600 MHz, $CDCl_3$, 25 °C) spectrum of **TFTMA**.

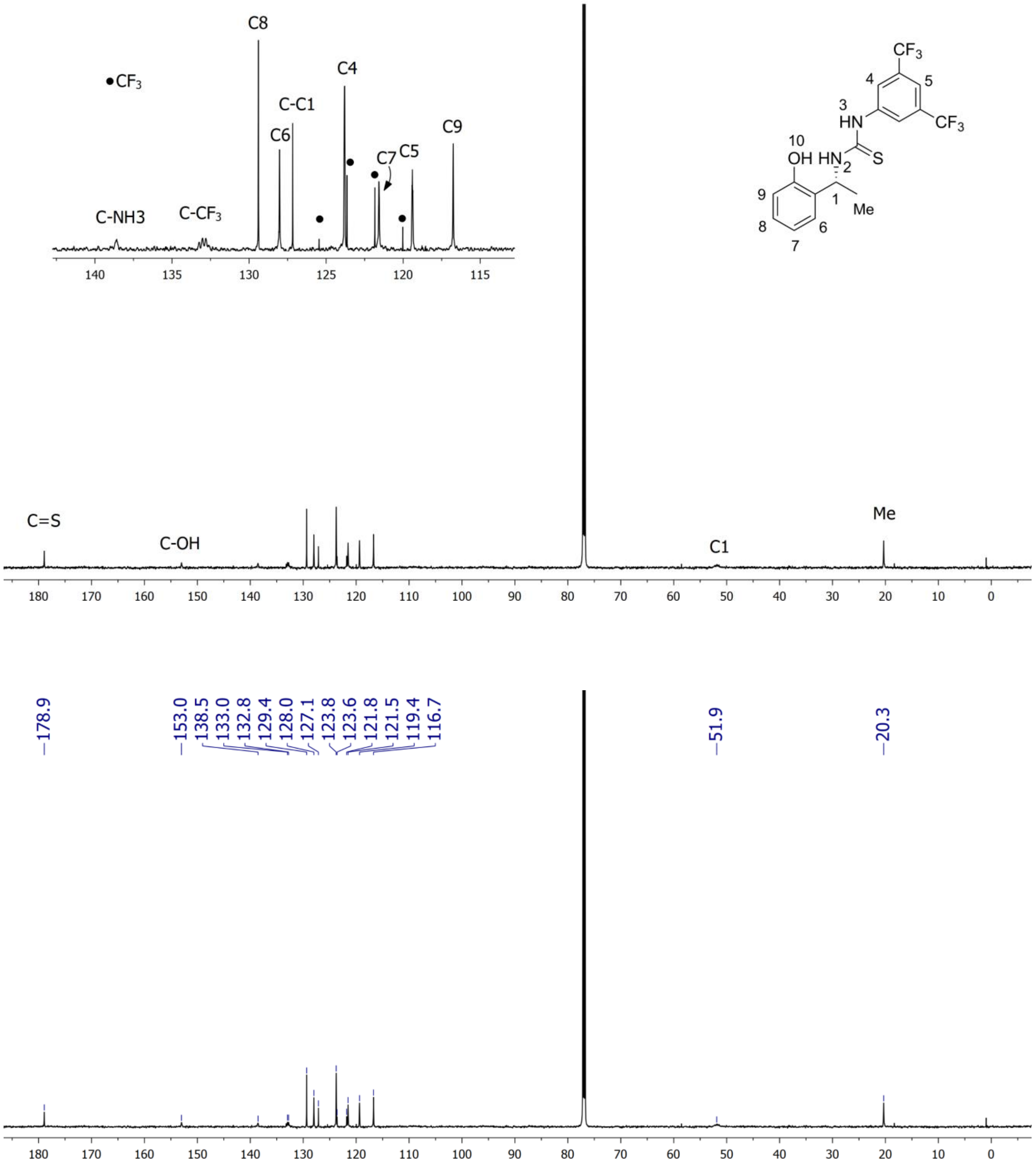

Figure S19. $^{13}C\{^{1}H\}$ NMR (150 MHz, $CDCl_3$, 25°C) spectrum of **TFTMA**.

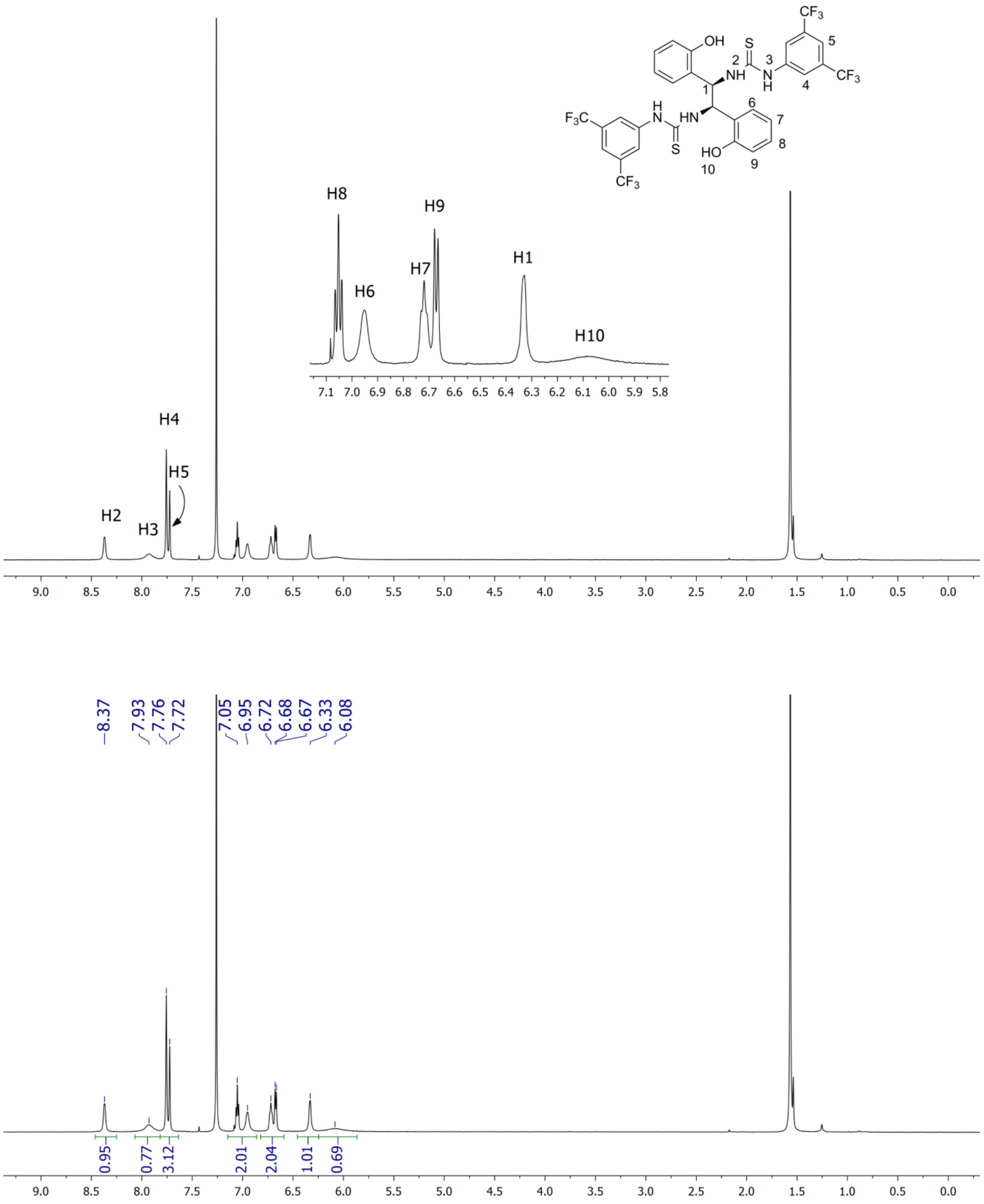

Figure S20. ^{1}H NMR (600 MHz, $CDCl_3$, 25°C) spectrum of **TFTDA.**

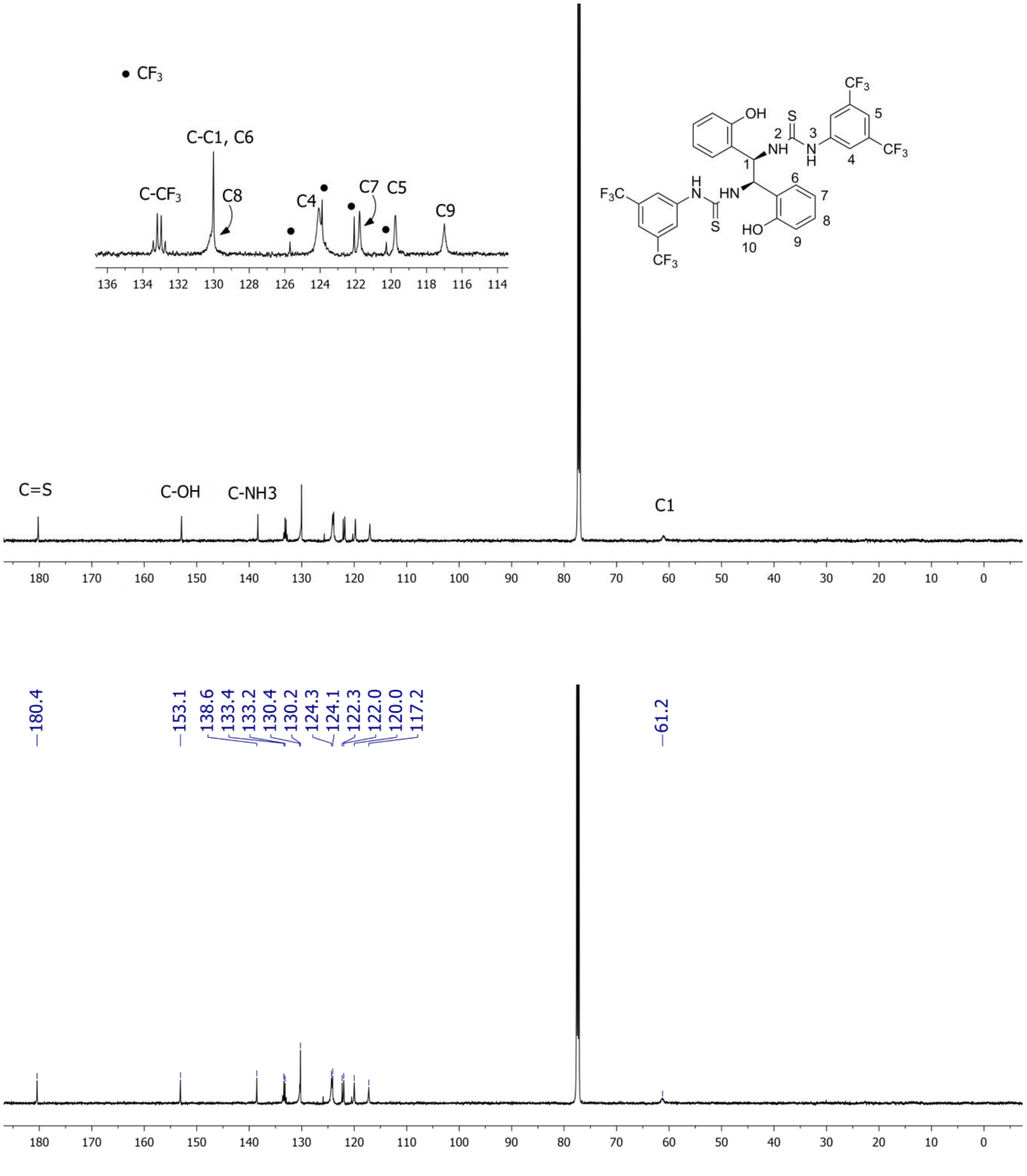

Figure S21. $^{13}C\{^{1}H\}$ NMR (150 MHz, $CDCl_3$, 25°C) spectrum of **TFTDA.**
